# *Pseudomonas aeruginosa* supports the survival of *Prevotella melaninogenica* in a cystic fibrosis lung polymicrobial community through metabolic cross-feeding

**DOI:** 10.1128/mbio.01594-25

**Published:** 2025-09-12

**Authors:** Bassam El Hafi, Fabrice Jean-Pierre, George A. O’Toole

**Affiliations:** 1 Department of Microbiology and Immunology, Geisel School of Medicine at Dartmouth 12285, Hanover, New Hampshire, USA; Gulbenkian Institute for Molecular Medicine, Oeiras, Portugal

**Keywords:** *Pseudomonas*, *Prevotella*, metabolism, polymicrobial, cross-feeding, cystic fibrosis, acetate, succinate

## Abstract

Cystic fibrosis (CF) is a multi-organ genetic disorder that affects more than 100,000 individuals worldwide. Chronic respiratory infections are among the hallmark complications associated with CF lung disease, and these infections are often due to polymicrobial communities that colonize the airways of persons with CF (pwCF). Such infections are a significant cause of morbidity and mortality, with studies indicating that pwCF who are co-infected with more than one organism experience more frequent pulmonary exacerbations, leading to a faster decline in lung function. Previous work established an *in vitro* CF-relevant polymicrobial community model composed of *P. aeruginosa*, *S. aureus*, *S. sanguinis*, and *P. melaninogenica. P. melaninogenica* cannot survive in monoculture in this model. In this study, we leverage this model to investigate the interactions between *P. aeruginosa* and *P. melaninogenica*, allowing us to understand the mechanisms by which the two microbes interact to support the growth of *P. melaninogenica* specifically in the context of the polymicrobial community. We demonstrate a cross-feeding mechanism whereby *P. melaninogenica* metabolizes mucin into short-chain fatty acids that are, in turn, utilized by *P. aeruginosa* and converted into metabolites (succinate, acetate) that are cross-fed to *P. melaninogenica*, supporting its survival in the CF lung-relevant model. This work highlights the potential metabolic interactions among microbes in CF infections.

**IMPORTANCE:**

Polymicrobial interactions impact disease outcomes in pwCF who suffer from chronic respiratory infections. Previous work established a CF-relevant polymicrobial community model that allows experimental probing of these microbial interactions to achieve a better understanding of the factors that govern the mechanisms by which CF lung microbes influence each other. In this study, we investigate the interaction between *P. aeruginosa* and *P. melaninogenica*, which are two highly prevalent and abundant CF lung microbes. We uncover a mechanism that involves complex cross-feeding between *P. aeruginosa* and *P. melaninogenica* to support the growth of the latter.

## INTRODUCTION

Cystic fibrosis (CF) is a genetic disorder that affects multiple organs in the human body, including the lungs, gut, pancreas, and kidneys (1–5). CF stems from a mutation in the cystic fibrosis transmembrane conductance regulator (CFTR) gene, leading to a dysfunctional CFTR ion channel, which results in the accumulation of thickened mucus in the airway (3–5). In the lungs, the mucus accumulation can lead to the partial or complete obstruction of the airways (3, 4). In fact, more than 80% of CF-related mortality before the onset of the newest therapies was due to lung disease characterized by chronic airway infections and related inflammation (6). It has now been recognized in the literature that persons with CF (pwCF) are often colonized by multiple microorganisms concurrently, establishing polymicrobial communities in their lungs. One feature of the polymicrobial nature of the CF lung is that the microbial community changes over time (7). Moreover, it has been observed that the CF polymicrobial community can be dominated by *Pseudomonas*, *Streptococcus*, and/or the aerotolerant anaerobic *Prevotella* (8–10). This finding suggests that the CF microbes influence the existence of each other *in vivo*. Therefore, understanding the mechanisms by which these organisms interact can explain the establishment, maintenance, and changes observed for the CF microbial community in the airway.

Previous work from our group aimed to develop an *in vitro*, CF-relevant, lung polymicrobial biofilm community model to represent the lung polymicrobial diversity among pwCF. To that end, publicly available 16S rRNA sequencing data retrieved from more than 160 clinical CF sputum samples were analyzed to identify the microbial genera that had the highest prevalence and highest abundance among pwCF and could be linked to CF disease respiratory outcomes (10, 11). The representative community is composed of *Pseudomonas aeruginosa*, *Staphylococcus aureus*, *Streptococcus sanguinis*, and *Prevotella melaninogenica* (10, 11). To translate this community to an experimental mixed-culture system, mucin-containing artificial sputum medium (ASM) (12, 13) was utilized under anoxic conditions at 37°C to grow these organisms and study their behavior in mono- and mixed-cultures (11). The rationale behind using ASM anoxically is that the medium nutritionally mimics the CF lung environment (13) and recapitulates the transcriptional profile of *P. aeruginosa* observed in the sputum (14–16). Furthermore, it has been previously demonstrated that the thick mucus lining the CF lung airways creates regions of anoxia (17).

In our previous study, we noted that *P. melaninogenica* could not be recovered when grown as a monoculture in mucin-containing ASM under anoxic growth conditions. By contrast, *P. melaninogenica* was found to be viable when co-cultured with *P. aeruginosa*, *S. aureus*, and *S. sanguinis* as a multi-species mixed culture (11). Such a finding was also consistent with previous *in silico* community modeling that predicted a low growth rate of *P. melaninogenica* in the CF lung environment (8). The mechanism whereby *P. melaninogenica* can grow in the community, but not in monoculture, has not been explored. Here, we describe a series of experimental studies that support a model whereby metabolic cross-feeding by another member of the community, *P. aeruginosa*, allows for the survival and growth of *P. melaninogenica* in the mixed community.

## RESULTS

### *P. aeruginosa* can support the viability of *P. melaninogenica* in a CF lung polymicrobial community model

Previous work from our group that aimed to develop an *in vitro*, CF-relevant, lung polymicrobial community model reported that *P. melaninogenica* could not be recovered as a monoculture in mucin-containing ASM under anoxic growth conditions. However, *P. melaninogenica* colony-forming units (CFUs) reached ~5 × 10^6^ when this microbe was cultured with *P. aeruginosa*, *S. aureus*, and *S. sanguinis* in a mixed culture (11). This finding aligned with previous *in silico* CF polymicrobial community simulations that predicted the single species growth rate of *P. melaninogenica* in the CF lung environment to be 2- to 2.5-fold lower than other CF-relevant bacteria (8). In addition, modeling using genome-scale metabolic reconstructions predicted that *P. aeruginosa* was the only member of the community to cross-feed *P. melaninogenica*; the predicted cross-fed metabolite was D-lactate. Meanwhile, *P. melaninogenica* was predicted to cross-feed alanine, aspartate, and formate to *P. aeruginosa* (8). These modeling studies predicted that *P. melaninogenica* grows more robustly in a mixed community and could participate in metabolic cross-feeding. Together with the observation that *P. melaninogenica* grew in the community but not in monoculture in our system, this prompted us to better understand the mechanisms of predicted and observed enhanced *P. melaninogenica* growth in the community.

To determine whether a specific member within the community was responsible for the *P. melaninogenica* growth phenotype, we co-cultured *P. melaninogenica* with the other organisms of the community in different combinations using mucin-containing ASM (11, 12) under anoxic growth conditions for 24 hours. Following incubation, the CFUs of *P. melaninogenica* were determined by serially diluting and spotting the biofilm fraction of each culture condition on the appropriate selective medium, then counting the resulting viable bacteria.

**Fig 1:**
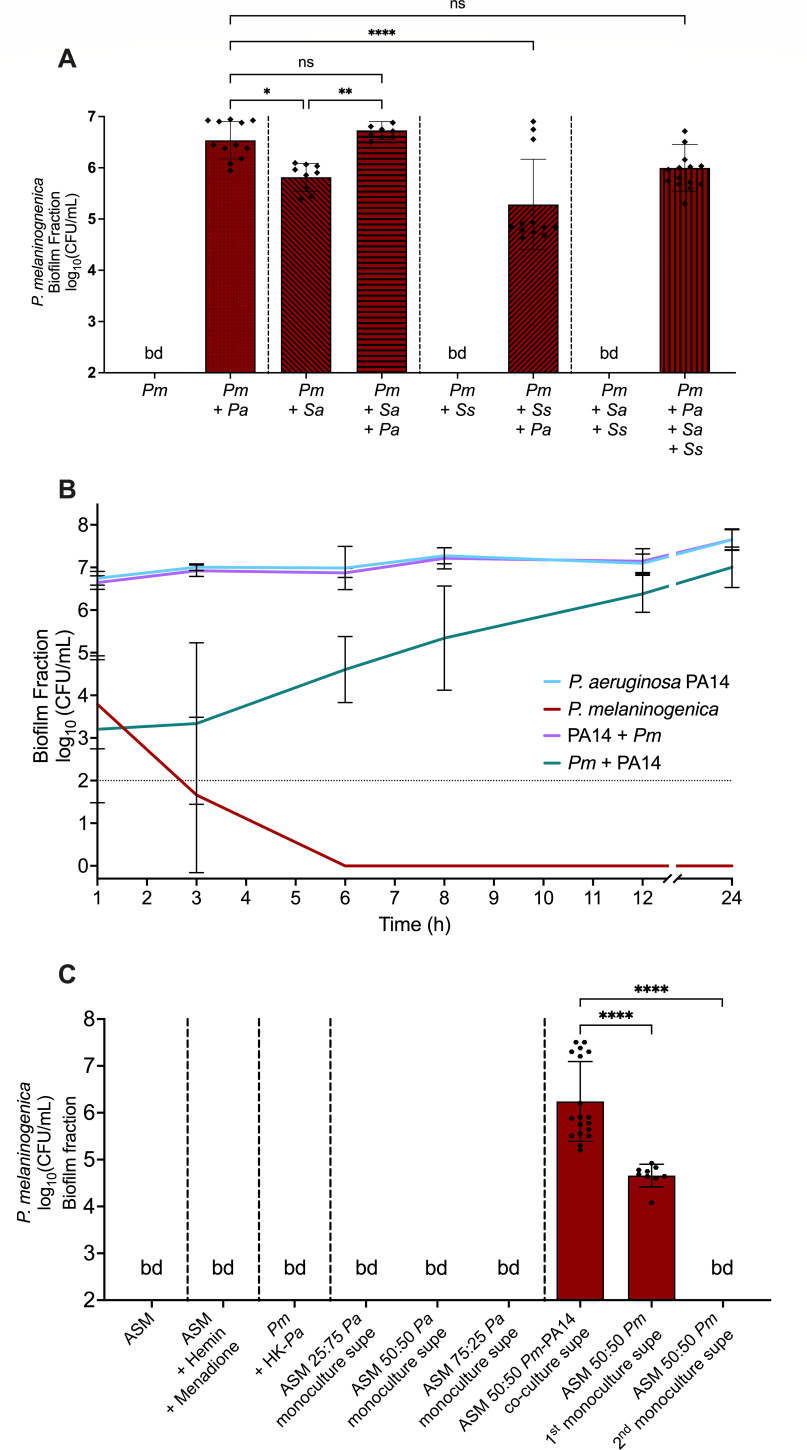
The growth of *P. melaninogenica* in co-cultures is enhanced in the presence of *P. aeruginosa*. (**A**) The co-culture of *P. melaninogenica* ATCC 25845 (*Pm*) with *P. aeruginosa* PA14 (*Pa*), *S. sanguinis* SK36 (*Ss*), and *S. aureus* Newman (*Sa*). All cultures were performed using mucin-containing ASM under anoxic growth conditions at 37°C for 24 hours. The CFUs derived from the biofilm fractions of the co-cultures are plotted. Statistical significance was calculated using ordinary one-way analysis of variance (ANOVA) with Tukey’s multiple comparisons test, *P < 0.05, **P < 0.005, ****P < 0.0001; ns, no significance. (**B**) A growth curve of *P. aeruginosa* PA14 and *P. melaninogenica* (*Pm*) CFUs in the biofilm fractions of their mono and co-cultures using ASM under anaerobic growth conditions at 37°C for 24 hours. The growth of *P. aeruginosa* is independent of the presence or absence of *P. melaninogenica*. The growth of *P. melaninogenica* depends on the presence of *P. aeruginosa*. Note that the growth of *P. melaninogenica* in co-culture with *P. aeruginosa* starts following a 6 h lag period. (**C**) The monoculture of *P. melaninogenica* (*Pm*) in mucin-containing ASM supplemented with hemin and menadione, heat-killed *P. aeruginosa* (HK-*Pa*) cells, or the spent ASM supernatants of *P. aeruginosa* monoculture (*Pa*), *P. aeruginosa-P. melaninogenica* co-culture (*Pa-Pm*), or *P. melaninogenica* (*Pm*) monocultures at different ratios with ASM. All cultures were performed under anoxic growth conditions at 37°C for 24 hours. The CFUs derived from the biofilm fractions are plotted. Statistical significance was calculated using ordinary one-way analysis of variance (ANOVA) with Tukey’s multiple comparisons test, ****P < 0.0001.

We observed that *P. aeruginosa* alone was sufficient to promote the growth of *P. melaninogenica* to the same extent as in a four-species mixture (Fig. 1A). The co-culture of *S. aureus* and *P. melaninogenica* resulted in the growth of the latter to a significantly lesser extent than with *P. aeruginosa*, and the addition of *P. aeruginosa* to that dual culture further enhanced the growth of *P. melaninogenica* (Fig. 1A, compare *Pm + Sa* to *Pm + Sa +* Pa). In comparison, *S. sanguinis* did not support *P. melaninogenica* in pairwise cultures nor in three-way cultures with *S. aureus*, indicating that *S. sanguinis* might be antagonistic to *P. melaninogenica* (Fig. 1A, *Pm + Ss* and *Pm + Ss +* Sa). However, the addition of *P. aeruginosa* to the *S. sanguinis-P. melaninogenica* dual culture, as well as the *S. aureus-S. sanguinis-P. melaninogenica* triple culture, rescued the growth of *P. melaninogenica* after 24 hours, demonstrating that *P. aeruginosa* supports, as well as likely protecting *P. melaninogenica* from microbial antagonism, in a mixed community (Fig. 1A). On the other hand, there were only modest (<0.5 log_10_) differences in the growth of *P. aeruginosa*, *S. aureus*, and *S. sanguinis* in the various co-culture combinations (Fig. S1A).

To understand the dynamics of the interaction between *P. aeruginosa* and *P. melaninogenica*, we conducted a time course assay of the monocultures and co-culture of both organisms in mucin-containing ASM under anoxic growth conditions, recording the CFU of the biofilm fractions of the cultures at the 1, 3, 6, 8, 12, and 24 hour time points. We observed that *P. aeruginosa* was not affected by the presence of *P. melaninogenica* since the growth curve of *P. aeruginosa* was nearly identical in both mono- and co-culture conditions, reaching ~10^8^ CFU/mL by the 24 h time point (Fig. 1B). By contrast, *P. melaninogenica* was not able to survive the monoculture condition as its viable population decreased over time until it was no longer detectable at the 6 h time point. However, in co-culture with *P. aeruginosa*, *P. melaninogenica* appeared to require a ~ 6 hour adjustment or lag period before steadily growing over the next 18 hours to ~10^6^ CFU/mL (Fig. 1B). In addition, the interaction between *P. aeruginosa* and *P. melaninogenica* was also not restricted to the biofilm fraction of the co-culture since the growth curves of *P. aeruginosa* and *P. melaninogenica* in the planktonic fraction of both mono- and co-culture conditions mirrored those in the biofilm fraction (Fig. S1B).

To determine whether *P. aeruginosa* was aiding in the recovery of *P. melaninogenica* through secreted products, we grew *P. melaninogenica* as a monoculture in mucin-containing ASM supplemented with hemin + menadione, with heat-killed *P. aeruginosa* cells, or with different ratios of spent cell-free supernatant of *P. aeruginosa* grown as a monoculture in ASM + mucin. *P. melaninogenica* could not be detected under any of these conditions (Fig. 1C). These data indicate that simple supplements, non-viable bacteria, or the secreted products of a *P. aeruginosa* monoculture cannot sufficiently support *P. melaninogenica* in this medium.

Next, we prepared a filter-sterilized spent supernatant from a *P. aeruginosa-P. melaninogenica* co-culture grown in ASM + mucin. This supernatant allowed for the successful recovery of *P. melaninogenica* in monoculture (Fig. 1C, ASM 50:50 *Pa-Pm* co-culture supe). Following the protocol illustrated in Fig. S1C, we then prepared supernatant from the monoculture of *P. melaninogenica* grown in the spent supernatant derived from the *P. aeruginosa-P. melaninogenica* co-culture in ASM + mucin, then repeated the monoculture growth of *P. melaninogenica*. We once again detected *P. melaninogenica* CFUs, although the final values were significantly reduced (Fig. 1C, ASM 50:50 *Pm* 1st monoculture supe). We repeated this process by generating a supernatant from the monoculture of *P. melaninogenica* in the “ASM 50:50 *Pm* 1st monoculture supe,” and used the new supernatant to test for the growth of *P. melaninogenica* in monoculture; however, this microbe was undetectable in this final condition (Fig. 1C, ASM 50:50 2nd *Pm* monoculture supe). On the other hand, we see no difference in the CFUs of *P. aeruginosa* under any of these culture conditions (Fig. S1D). These data imply that secreted products can only rescue *P. melaninogenica* when *P. aeruginosa* and *P. melaninogenica* are grown together, indicating that the cross-feeding requires microbial interactions. Finally, the observation that repeated passages in a spent supernatant cannot effectively sustain the detection of *P. melaninogenica* in monoculture indicates that the *P. aeruginosa*-mediated growth of *P. melaninogenica* requires continuous co-culture of the two organisms. Together, these observations demonstrate the dynamic nature of the interaction between these organisms and support a model whereby *P. melaninogenica* benefits from the presence of *P. aeruginosa* in co-culture via shared secreted products.

### A genetic screen identifies *P. aeruginosa* mutants unable to fully support *P. melaninogenica* growth when co-cultured in mucin-containing ASM

**Fig 2:**
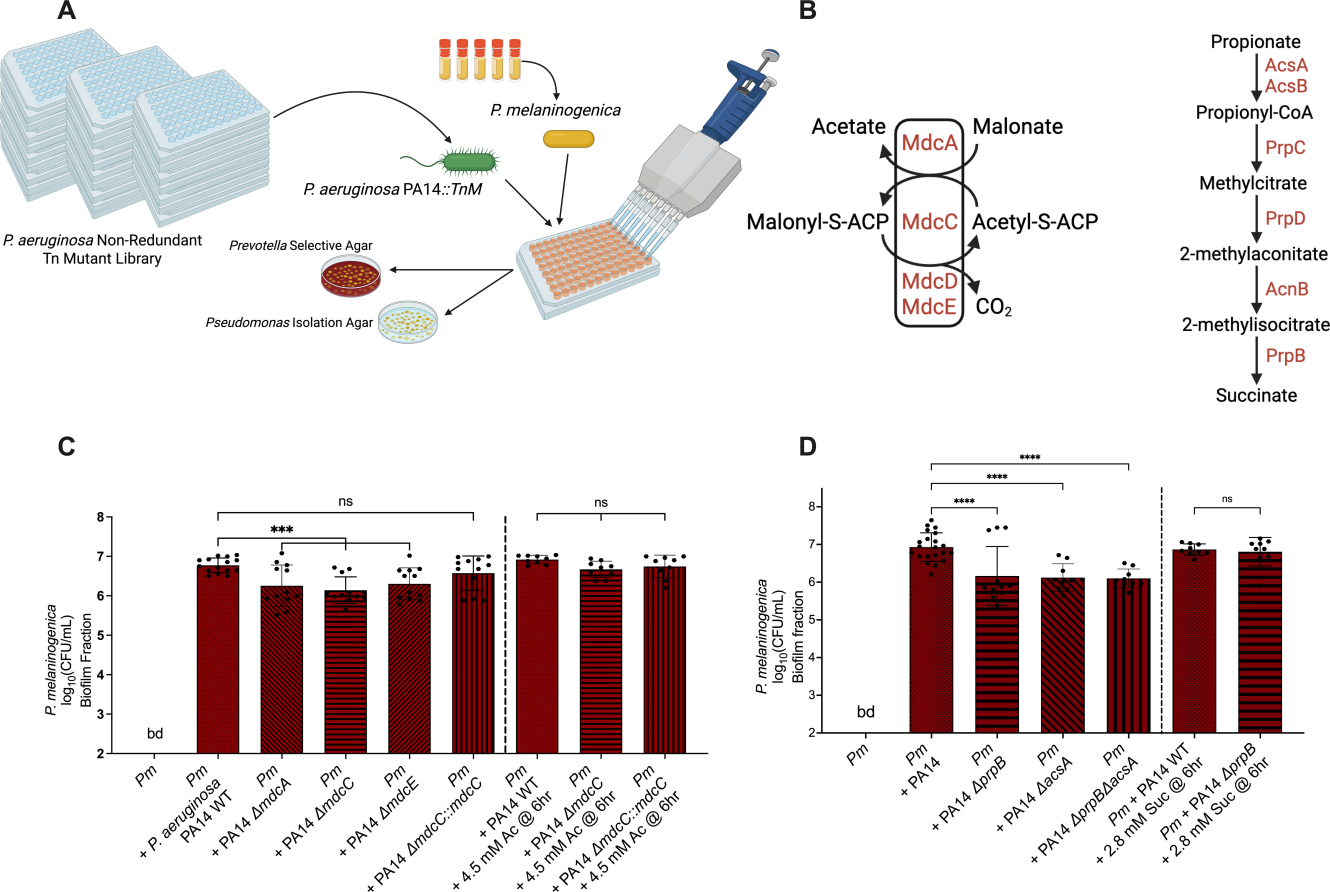
*P. aeruginosa* genetic screen identifies carbon metabolism pathways implicated in the interaction with *P. melaninogenica*. (**A**) A schematic diagram of the *P. aeruginosa* PA14 non-redundant transposon mutant library screen in co-culture with *P. melaninogenica*. Figure designed using BioRender. (**B**) Pathways showing the metabolism of malonate (19, 20) and propionate (19) into acetate and succinate, respectively, with selected genes involved in the metabolism of these carbon sources. (**C and D**) Bar plots of the CFUs derived from the biofilm fractions of *P. melaninogenica* (*Pm*). All cultures were performed using mucin-containing ASM under anaerobic growth conditions at 37°C for 24 hours. Statistical significance was calculated using ordinary one-way analysis of variance (ANOVA) with Tukey’s multiple comparisons test. (**C**) The pairwise co-culture of *P. melaninogenica* with WT *P. aeruginosa* PA14 and the Δ*mdcA*, Δ*mdcE,* and Δ*mdcC* mutants, as well as the Δ*mdcC::mdcC* complemented strain, with and without the supplementation of 4.5 mM of acetate at 6 h, ***P < 0.05; ns, no significance. The concentrations of acetate used here were selected based on the data presented in Fig. 4A. (**D**) The pairwise co-culture of *P. melaninogenica* (*Pm*) with WT *P. aeruginosa* PA14 and Δ*prpB*, Δ*acsA*, and Δ*prpB*Δ*acsA* mutants, with and without the supplementation of 2.8 mM of succinate at 6 h, ****P < 0.0001; ns, no significance. The concentrations of succinate used here were selected based on the data presented in Fig. 4A.

We next sought to investigate the mechanisms that govern the interaction between *P. aeruginosa* and *P. melaninogenica* using a genetic approach. To achieve this goal, we screened the *P. aeruginosa* PA14 non-redundant transposon mutant library (18) in co-culture with *P. melaninogenica,* growing the organisms on mucin-containing ASM under anoxic conditions, to identify *P. aeruginosa* PA14 transposon mutants that were incapable of, or showed a reduced ability for, supporting the growth of *P. melaninogenica* (Fig. 2A). We reasoned that this approach would allow us to identify genetic determinants of *P. aeruginosa* that are required for its ability to promote the growth of *P. melaninogenica* in co-culture.

Mutant candidates that were identified in the primary screen were subjected to a second and third round of re-testing to generate the final list of *P. aeruginosa* PA14 transposon mutants that showed reduced ability to support the growth of *P. melaninogenica* in co-culture compared to the wild-type (WT) *P. aeruginosa* PA14 in mucin-containing ASM during anaerobic growth (Table S1). Many of the identified *P. aeruginosa* PA14 mutants from our screen had genes of unknown function. However, a few mutations were in genes with defined functions, such as *sppR*, which encodes the TonB-dependent receptor SppR. The *sppR* gene is co-transcribed with the *spp* operon, which is responsible for the expression of the Spp transporter, involved in xenosiderophore uptake (21, 22). Another mutation mapped to the *cupD4* gene, which encodes an adhesin in the CupD fimbrial assembly (21, 23).

A number of mutations were in genes involved in either carbon metabolism or amino acid biosynthesis and metabolism, namely (i) *mdcE*, which encodes a subunit of malonate decarboxylase (19, 21, 24, 25), (ii) *prpR*, which encodes the transcriptional activator of the *prp* genes that encode the enzymes responsible for metabolizing propionate to succinate (19, 21), (iii) *hutU*, which is part of the histidine utilization locus and encodes urocanate hydratase that converts urocanate into imidazolone propionate as part of the histidine catabolism pathway of *Pseudomonas fluorescens* (21, 26), and (iv) PA14_38140, which is the ortholog of the *pauA4* gene of *P. aeruginosa* PAO1 (21) that encodes glutamylpolyamine synthetase involved in polyamine metabolism (27).

Identifying genes in our genetic screen with defects in these metabolic pathways is consistent with metabolic modeling studies that were previously conducted by our group (8, 11). Those modeling efforts identified several organic acids and amino acids that were predicted to be cross-fed between the members of the CF-relevant polymicrobial community. Therefore, we decided to focus on select *P. aeruginosa* PA14 metabolism-related pathways identified by the genetic screen to better understand their role in the interaction between *P. aeruginosa* and *P. melaninogenica*.

### Genetic and feeding studies support the conclusion that acetate and succinate contribute to the growth of *P. melaninogenica* when co-cultured with *P. aeruginosa* in mucin-containing ASM

In *P. aeruginosa*, malonate is decarboxylated to acetate via malonate decarboxylase (Fig. 2B, left), which is composed of multiple subunits encoded by the *mdcABCDEGHLM* operon. The *mdcABCDEGH* genes encode functional subunits of the Mdc enzyme complex, and the *mdcLM* genes encode the malonate transporter (25). Propionate, on the other hand, is metabolized via the methylcitrate pathway (Fig. 2B, right), which is composed of a number of intermediary steps, starting with the conversion of propionate to propionyl-CoA via acetyl-CoA synthetase, encoded by *acsA*, and ending with the conversion of 2-methylisocitrate into succinate via 2-methylisocitrate lyase, encoded by the *prpB* gene (19, 21, 28–30).

To examine the involvement of malonate and propionate metabolism pathways in the interaction between *P. aeruginosa* and *P. melaninogenica*, we acquired previously reported *P. aeruginosa* PA14 strains carrying deletion mutations of three different *mdc* genes that encode the active site subunits of Mdc (25), as well as the deletion mutants of the *acsA* and *prpB* genes, which, respectively, encode AcsA and PrpB, required for the metabolism of propionate to succinate (31). We co-cultured the selected *P. aeruginosa* mutants with *P. melaninogenica* in mucin-containing ASM under anoxic growth conditions, then counted the resulting CFUs of each organism by plating on selective media and compared those results to the growth of *P. melaninogenica* in co-culture with WT *P. aeruginosa* PA14.

The co-culture of *P. melaninogenica* with the *P. aeruginosa* PA14 Δ*mdcA,* D*mdcC,* and Δ*mdcE* mutants resulted in a significant, ~10-fold decrease in viable *P. melaninogenica* counts when compared to its co-culture with WT *P. aeruginosa* PA14 (Fig. 2C). In addition, co-culturing *P. melaninogenica* with the *P. aeruginosa* PA14 Δ*mdcC::mdcC,* a complemented strain, restored the viable counts of *P. melaninogenica* to a level not significantly different from WT *P. aeruginosa* PA14 (Fig. 2C).

We then asked whether supplementing the end-product of malonate metabolism (i.e., acetate) would reverse the growth defect of *P. melaninogenica* when co-cultured with the *mdc* mutants. Interestingly, adding 4.5 mM of acetate to the *P. melaninogenica-P. aeruginosa* PA14 Δ*mdcC* co-culture at the 6 h time point, but not at the start of the experiment (t = 0, not shown), restored the growth of *P. melaninogenica* to levels observed in co-culture with WT *P. aeruginosa* PA14 (Fig. 2C, right three bars). We used 4.5 mM of acetate and the concentration of succinate used in the next section, based on data presented below (Fig. 4). The 6 h time point was selected because, as we noted earlier, *P. melaninogenica* growth in co-culture with WT *P. aeruginosa* PA14 appeared to increase starting at the 6 h time point in the time course assay (Fig. 1B).

Similar to our observations analyzing the *mdc* mutants, the co-culture of *P. melaninogenica* with *P. aeruginosa* PA14 Δ*prpB*, Δ*acsA*, and Δ*prpB*D*acsA* mutants resulted in a significant, ~10-fold decrease in the viable counts of *P. melaninogenica* compared to its growth with WT *P. aeruginosa* PA14 (Fig. 2D). Moreover, the supplementation of the *P. aeruginosa* PA14 Δ*prpB* and *P. melaninogenica* co-culture with 2.8 mM succinate, the metabolic end-product of propionate catabolism via the methyl citrate cycle, at the 6 h timepoint also restored the growth of *P. melaninogenica* to levels observed in co-culture with WT *P. aeruginosa* PA14 (Fig. 2D, right two bars). The growth of wild-type *P. aeruginosa* PA14, as well as the mutants tested here, was not substantially different under any of these conditions (Fig. S2A and B).

We next tested whether the addition of 4.5 mM acetate, 2.8 mM succinate, 5 mM malonate, 5 mM propionate, and 5 mM fumarate, separately or as a cocktail, to mucin-containing ASM would be sufficient to rescue *P. melaninogenica* monocultures. We chose the first four metabolites because they represent the substrate and product of the Mdc and Prp pathways. Fumarate was added because it has been previously reported to be a terminal electron acceptor in *Prevotella* spp. (32). *P. melaninogenica* remained unrecoverable in monoculture after 24 hours of anoxic incubation regardless of the metabolite supplementation (Fig. S2C). The concentrations of acetate and succinate were selected based on their initial levels measured in the *P. aeruginosa-P. melaninogenica* co-culture (see Fig. 4), while the concentrations of malonate, propionate, and fumarate were chosen to closely match those of acetate and succinate.

**Fig 3:**
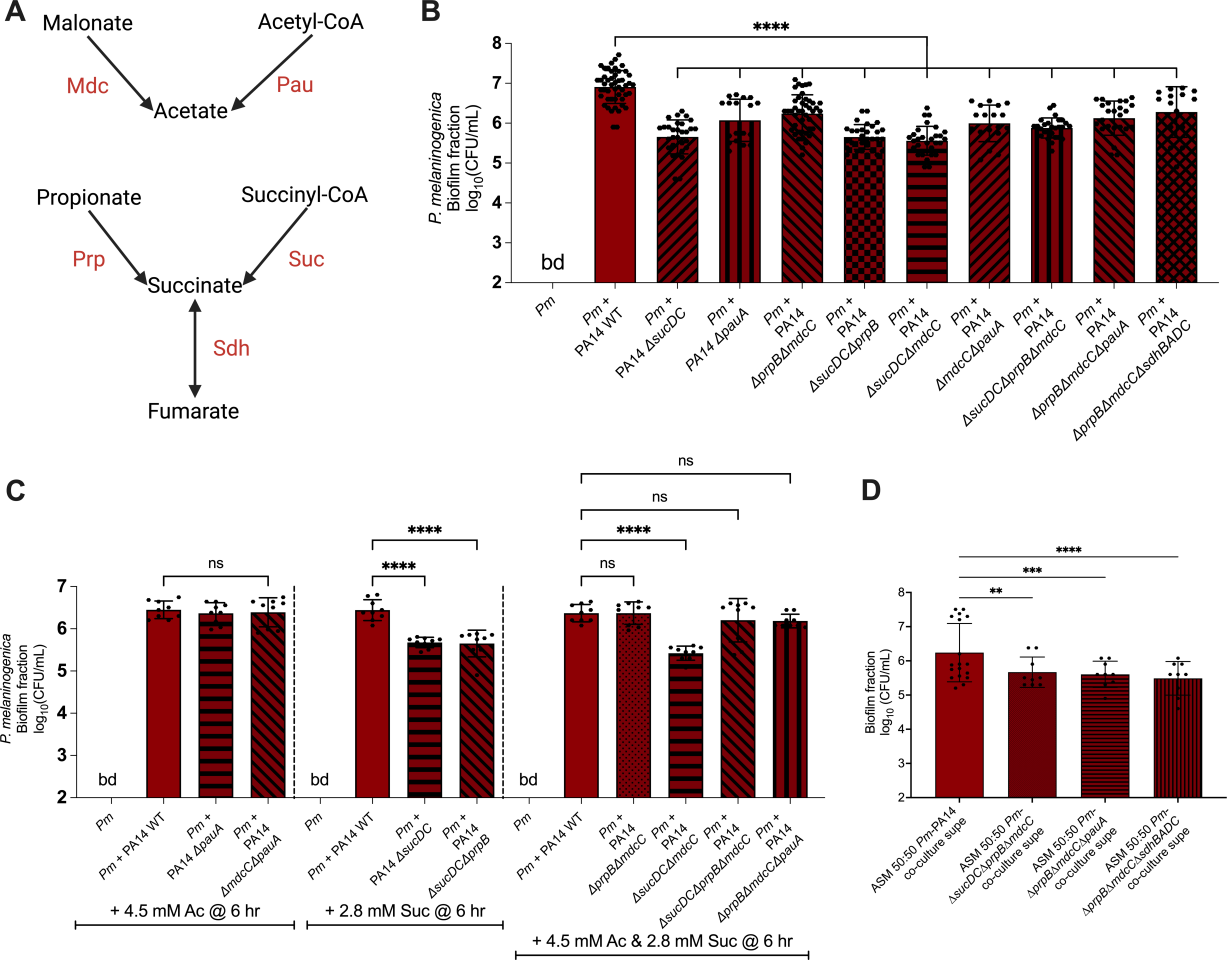
Additional metabolic pathways that generate acetate and succinate in *P. aeruginosa* are also implicated in the interaction with *P. melaninogenica*. (**A**) A schematic diagram indicating multiple metabolic pathways that generate acetate and succinate in *P. aeruginosa*. (**B and C**) Bar plots of the CFUs derived from the biofilm fractions of *P. melaninogenica* (*Pm*). All cultures were performed using mucin-containing ASM under anoxic growth conditions at 37°C for 24 hours. Statistical significance was calculated using ordinary one-way analysis of variance (ANOVA) with Tukey’s multiple comparisons test. (**B**) The co-culture of *P. melaninogenica* (*Pm*) with WT *P. aeruginosa* PA14 or the Δ*sucDC*, Δ*pauA*, Δ*prpB*Δ*mdcC*, Δ*sucDC*Δ*prpB,* Δ*sucDC*Δ*mdcC*, Δ*mdcC*Δ*pauA*, Δ*sucDC*Δ*prpB*Δ*mdcC*, Δ*sucDC*Δ*prpB*Δ*pauA*, and Δ*sucDC*Δ*prpB*Δ*sdhBADC* mutants, ****P < 0.0001. (**C**) Selected strains from panel B supplemented, as indicated, with acetate (left), succinate (middle), or both (right), ****P < 0.0001; ns, no significance. (**D**) The monocultures of Pm with the supernatants of PA14 Δ*sucDC*Δ*prpB*Δ*mdcC*, Δ*sucDC*Δ*prpB*Δ*pauA*, and Δ*sucDC*Δ*prpB*Δ*sdhBADC* as well as WT PA14 as control from co-cultures with *P. melaninogenica*. **P < 0.005; ***P < 0.0005; ****P < 0.0001.

To investigate the impact of disrupting additional metabolic pathways responsible for generating acetate and succinate in *P. aeruginosa* PA14 (Fig. 3A), we created single and combination deletion mutants in the ∆*mdcC* and ∆*prpB* mutant backgrounds. The genes deleted include *pauA*, which encodes pimeloyl-CoA synthetase and is involved in converting acetyl-CoA to acetate; the *sucDC* operon, which encodes succinyl-CoA synthetase that metabolizes succinyl-CoA to succinate; and the *sdhBADC* operon that encodes succinate dehydrogenase that converts fumarate to succinate (28–30). In total, nine additional deletion mutants were generated: *P. aeruginosa* PA14 ∆*pauA*, ∆*sucDC*, ∆*prpB*∆*mdcC*, ∆*sucDC*∆*prpB*, ∆*sucDC*∆*mdcC*, ∆*mdcC*∆*pauA*, ∆*sucDC*∆*prpB*∆*mdcC*, ∆*prpB*∆*mdcC*∆*pauA*, and ∆*prpB*∆*mdcC*∆*sdhBADC*.

Upon co-culturing *P. melaninogenica* with the additional PA14 mutants in mucin-containing ASM under anoxic growth conditions, we noted a significant, ~10- to 15-fold decrease in the recovery of *P. melaninogenica* compared to its co-culture with WT *P. aeruginosa* PA14 at the 24 h time point (Fig. 3B), with the largest effect being observed when the *sucDC* genes were deleted either as a single or combination mutant. Moreover, upon supplementing the co-cultures with acetate and/or succinate at the 6 h time point, depending on whether each individual or both metabolic pathways were disrupted, we observed that the growth of *P. melaninogenica* was fully rescued except in the co-cultures with PA14 ∆*sucDC*, ∆*sucDC*∆*prpB*, and ∆*sucDC*∆*mdcC*, which still showed a growth defect in co-culture (Fig. 3C). In addition, we observed that *P. melaninogenica* CFUs were significantly lower when grown as a monoculture in mucin-containing ASM supplemented 50:50 with the spent cell-free supernatant of *P. melaninogenica* in co-culture with either *P. aeruginosa* PA14 ∆*sucDC*∆*prpB*∆*mdcC*, ∆*prpB*∆*mdcC*∆*pauA*, or ∆*prpB*∆*mdcC*∆*sdhBADC* compared to WT PA14 (Fig. 3D). Lastly, it is important to note that the *P. aeruginosa* mutants used here showed no significant changes in viable counts when grown in mono- or co-cultures (Fig. S3A through D).

### Acetate and succinate are elevated in the *P. aeruginosa*-*P. melaninogenica* co-culture compared to monoculture

**Fig 4:**
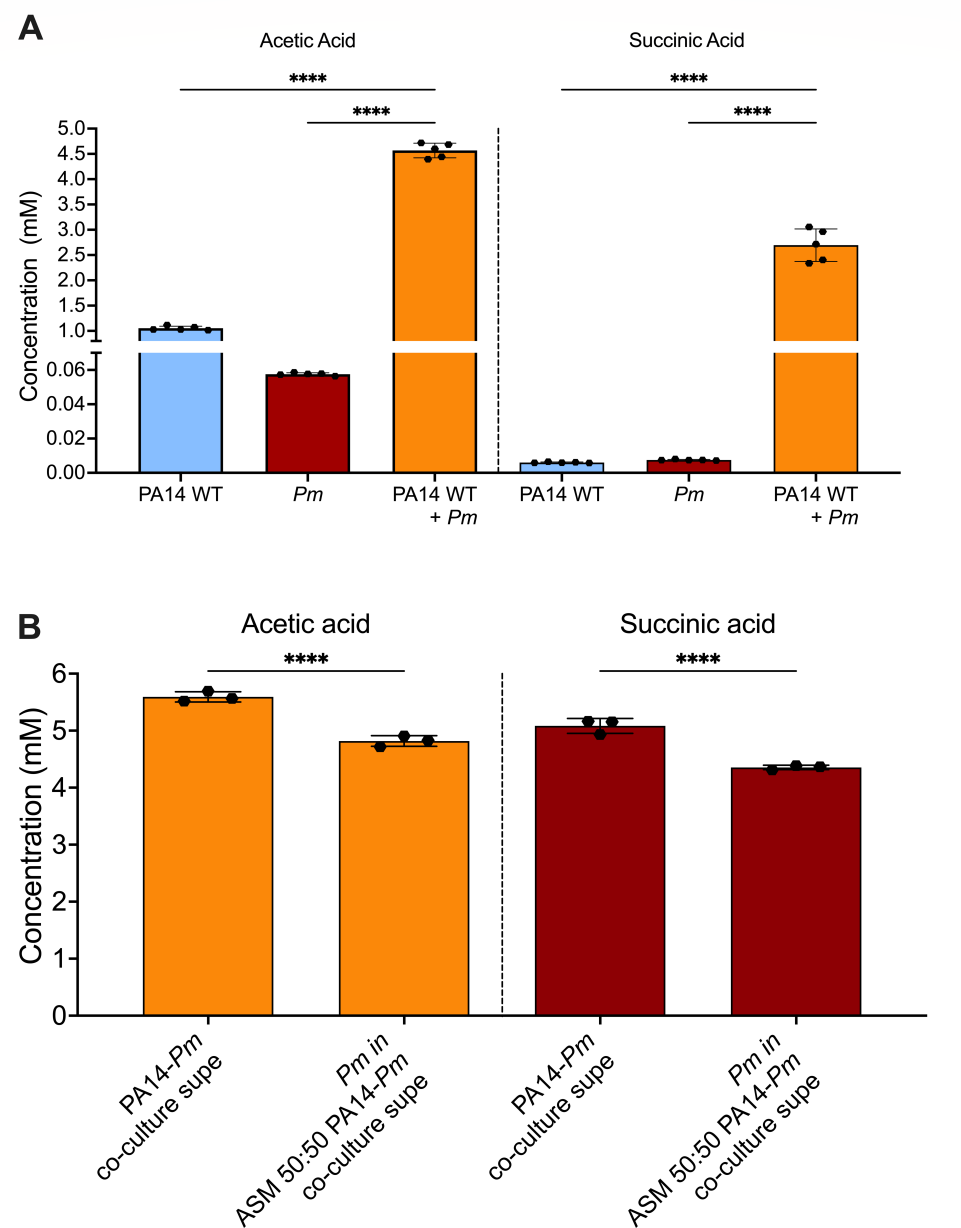
Acetate and succinate concentrations are significantly higher in *P. aeruginosa-P. melaninogenica* co-cultures compared to monoculture growth. (**A**) The concentrations of acetate and succinate as measured by GC-MS in the cell-free mono- and co-culture ASM + Mucin supernatants of WT *P. aeruginosa* PA14 and *P. melaninogenica* following a 24 h incubation under anoxic growth conditions at 37°C. (**B**) The concentrations of acetate and succinate as measured by GC-MS for supernatants prepared from a co-culture of *P. aeruginosa* and *P. melaninogenica* in ASM + mucin (left bars) or that the same filter-sterilized supernatants after incubation with *P. melaninogenica* for 24 h (right bars) under anaerobic conditions. Statistical significance was calculated for data in both panels using ordinary one-way analysis of variance (ANOVA) with Tukey’s multiple comparisons test, ****P < 0.0001.

Given that mutations in the pathways required for the metabolism of malonate and propionate by *P. aeruginosa* alter its interaction with *P. melaninogenica*, we sought to measure the concentration of these metabolites, as well as their respective products generated by the Mdc and Prp pathways, acetate and succinate. We employed GC-MS to quantify the concentrations of these metabolites at multiple time points over a 24 h period in the cell-free supernatants of the mono- and co-cultures of *P. aeruginosa* and/or *P. melaninogenica* in mucin-containing ASM under anoxic growth conditions. Presenting the data at the 24 h time point (Fig. 4A), we observed that propionate was undetectable in all culture conditions, and, unfortunately, we could not measure malonate using the method developed for these studies. The concentrations of acetate and succinate were 4.5 mM and 2.8 mM, respectively, at 24 h in co-culture conditions (Fig. 4A). Meanwhile, both metabolites were significantly lower in the monocultures of both *P. aeruginosa* and *P. melaninogenica*. The time-course measurements showed that these compounds appeared in the coculture by 6 h and were higher at the 24 h time point (Fig. S4). These data are consistent with the conclusion that acetate and succinate are only produced in appreciable amounts in the coculture, and this accumulation occurs largely at or after 6 h of co-incubation.

As described in Fig. 1C, we showed that *P. melaninogenica* could grow in monoculture when supplied with supernatant from a *P. aeruginosa-P. melaninogenica* co-culture but not a *P. aeruginosa* monoculture. Furthermore, we observed a reduction in *P. melaninogenica* viable counts with successive propagation in the co-culture supernatant. To test whether acetate and succinate might be involved in the support of *P. melaninogenica* growth in the *P. aeruginosa-P. melaninogenica* co-culture supernatant, we hypothesized that these metabolites should be reduced in concentration after the growth of *P. melaninogenica*. Indeed, we see that the levels of acetate and succinate are significantly reduced after *P. melaninogenica* is allowed to grow in the *P. aeruginosa-P. melaninogenica* co-culture supernatant (Fig. 4B).

### Carbon catabolite repression contributes to the *P. melaninogenica*-*P. aeruginosa* interaction through C_4_-dicarboxylate transport

Since multiple metabolic pathways were found to be implicated in the interaction between *P. aeruginosa* and *P. melaninogenica*, we sought to assess the effect of disrupting global metabolic pathways on the interaction between both organisms by targeting carbon catabolite repression (CCR) in *P. aeruginosa*. CCR is a post-transcriptional metabolic regulatory process that establishes a hierarchy of preference toward the consumption of carbon sources (33). The two-component signaling system CbrAB, as well as the catabolite repression control protein Crc, is critical in the *P. aeruginosa* CCR system (33). Therefore, we acquired previously reported *P. aeruginosa* PA14 Δ*cbrA,* Δ*cbrB,* and Δ*crc* mutants (34) and co-cultured these mutants with *P. melaninogenica*. There was a modest, but statistically significant, decrease in the growth of *P. melaninogenica* when co-cultured with the *P. aeruginosa* PA14 CCR mutants compared to WT (Fig. S5A). None of these mutants had a growth defect in monoculture at the same 24 h time point (Fig. S5B). Overall, defects in the CCR system of *P. aeruginosa* alter its interaction with *P. melaninogenica*.

We additionally investigated the involvement of a variety of CCR targets (35) in the interaction between *P. aeruginosa* and *P. melaninogenica* by co-culturing the latter with *P. aeruginosa* PA14 *dctA::Tn*M, ∆*phzA1*∆*phzA2*, ∆*mvfR*/*pqsR*, and ∆*pqsH* mutants. Only the mutation in the strain with a mutation in *dctA*, which encodes a C_4_-dicarboxylate transporter of fumarate, malate, and succinate (36), showed a significant reduction in *P. melaninogenica* viability compared to WT PA14 (Fig. S6A through C). These data are also consistent with *P. aeruginosa* requiring C_4_-dicarboxylates to effectively support the growth of *P. melaninogenica*.

### Mucin cannot support the growth of *P. aeruginosa* in our experimental system, consistent with previous findings

While we were able to measure the concentrations of acetate and succinate in the cell-free supernatants of our *in vitro* model as described in the previous section, the source of those compounds, as well as their respective malonate and propionate precursors, was still in question since they are not components of the culture medium we prepared. Therefore, we first hypothesized that perhaps mucin was metabolized to generate malonate and propionate, which was, in turn , utilized by *P. aeruginosa*.

**Fig 5:**
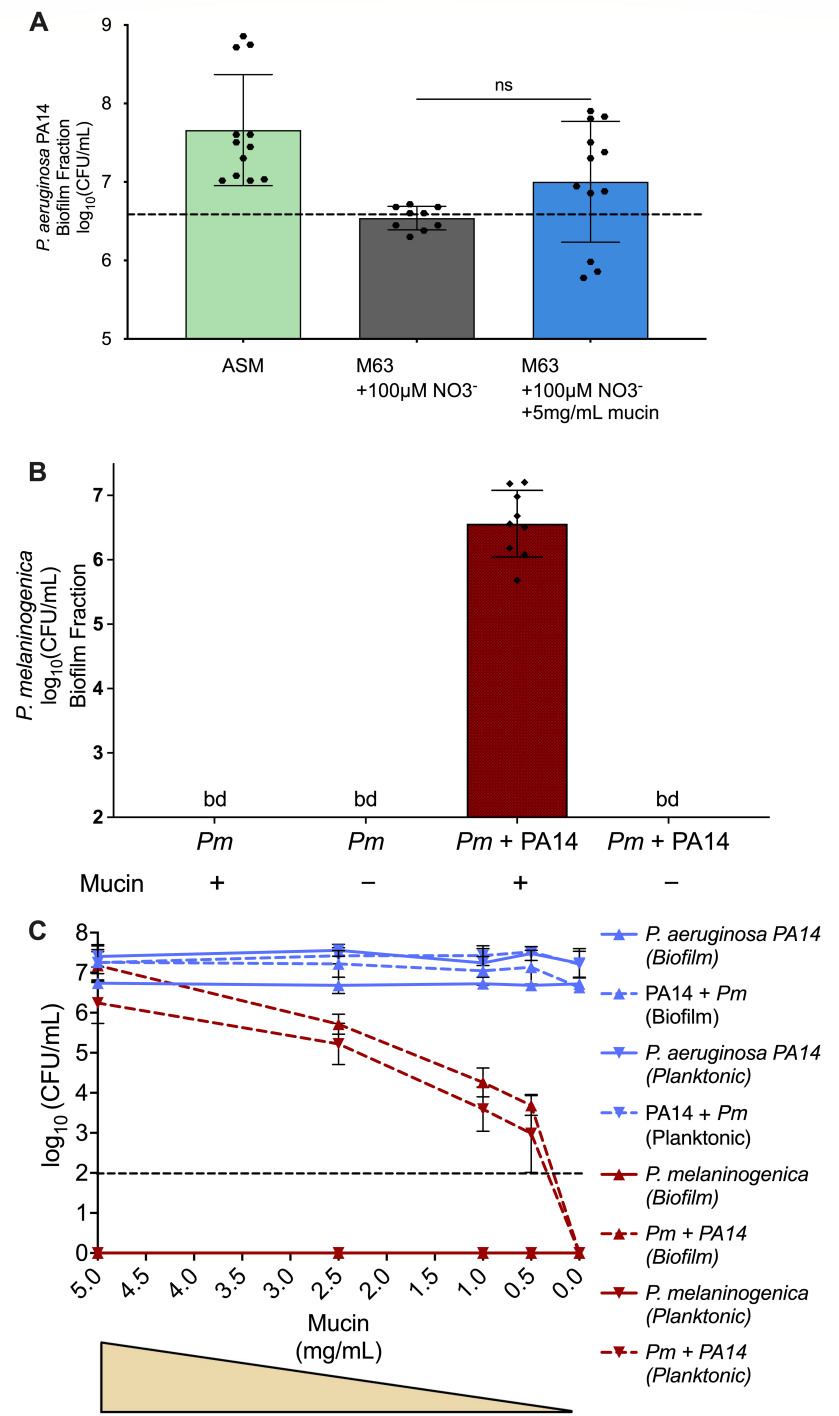
Mucin is required for *P. aeruginosa* promotion *of P. melaninogenica* growth. (**A**) The monoculture of *P. aeruginosa* PA14 in ASM compared to minimal medium supplemented with nitrate ± mucin as the main carbon source was conducted under anaerobic growth conditions at 37°C. The dashed line indicates the starting inoculum concentration of *P. aeruginosa*. The CFUs of the biofilm fraction of *P. aeruginosa* PA14 are plotted. Statistical significance was calculated using ordinary one-way analysis of variance (ANOVA) with Tukey’s multiple comparisons test. No statistical significance was found (ns, *P*-value > 0.05). (**B**) The mono- and co-culture of *P. melaninogenica* (*Pm*) with *P. aeruginosa* PA14 in the presence and absence of mucin in ASM. The CFUs of the biofilm fractions of *P. melaninogenica* are plotted from an experiment conducted under anaerobic growth conditions at 37°C for 24 hours. *P. aeruginosa* does not support the growth of *P. melaninogenica* in the absence of mucin. (**C**) The mono and co-cultures of *P. melaninogenica* (*Pm*) and *P. aeruginosa* PA14 in ASM with decreasing concentrations of mucin. The CFUs of both planktonic and biofilm fractions are plotted. The experiment was conducted under anaerobic growth conditions at 37°C for 24 hours. The survival of *P. melaninogenica* in co-culture with *P. aeruginosa* depends on mucin. The horizontal dashed line indicates the limit of detection.

Previous work by Flynn et al. (31) showed that *P. aeruginosa* cannot utilize mucin as a sole carbon source. We observed similar findings here by culturing WT *P. aeruginosa* PA14 anaerobically in M63 minimal medium with mucin as the sole carbon/energy source and nitrate as the electron acceptor and compared its biofilm fraction CFUs after 24 hours to those recorded from the culture in M63 minimal media lacking mucin, as well as to mucin-containing ASM as a positive control. We found that mucin, as a sole energy source, did not significantly promote the growth of *P. aeruginosa* in the minimal medium, since the viable counts remained at initial inoculum of ~10^6^ to 10^7^ CFU/mL (dotted line) after 24 hours, whether or not mucin was present (Fig. 5A). These results indicate that *P. aeruginosa* does not effectively utilize mucin as a carbon source; thus, it was unlikely able to metabolize mucin into the metabolic intermediates (i.e., malonate and propionate) implicated in its interaction with *P. melaninogenica*.

### Mucin is a critical factor mediating the *P. aeruginosa*-*P. melaninogenica* interaction under CF-like nutritional environments

To assess whether mucin is required for *P. aeruginosa* to support the growth of *P. melaninogenica* in our *in vitro* model, we grew the organisms in mono- and co-cultures in ASM with and without mucin, then enumerated the resulting biofilm fraction CFUs after 24 hours of anoxic incubation at 37°C. As expected, *P. melaninogenica* was not detectable when grown as a monoculture ± mucin (Fig. 5B). By contrast, *P. melaninogenica* was detectable when co-cultured with *P. aeruginosa* in mucin-containing ASM, as expected; however, *P. melaninogenica* growth was no longer observed in co-culture with *P. aeruginosa* in ASM lacking mucin (Fig. 5B). *P. aeruginosa* PA14 was recoverable in the biofilm fraction of both monocultures and co-cultures, with and without mucin. In monoculture, there was no significant difference in CFU ± mucin, while in co-culture, there was a significant but modest increase (<0.5 log_10_) in *P. aeruginosa* PA14 viable counts with mucin (Fig. S7). This observation demonstrated the necessity of mucin for the survival of *P. melaninogenica* in co-culture with *P. aeruginosa*.

To further investigate the dependence of *P. melaninogenica* on mucin in the co-culture medium, we anoxically grew the mono- and co-cultures of *P. aeruginosa* and *P. melaninogenica* in ASM with decreasing concentrations of mucin (5, 2.5, 1, 0.5, and 0 mg/mL), then plotted the resulting biofilm and planktonic CFUs at the 24 h timepoint. It was evident that the growth of WT *P. aeruginosa* PA14 was neither affected by the concentration of mucin nor by the presence of *P. melaninogenica* (Fig. 5C). Unsurprisingly, *P. melaninogenica* did not survive the monocultures in either biofilm or planktonic fractions regardless of the concentration of mucin (Fig. 5C). However, *P. melaninogenica* appeared to exhibit a dose-dependent response to the concentration of mucin in both biofilm and planktonic fractions of the co-culture, where the decrease in the concentration of mucin directly correlated with the decrease in the viability of *P. melaninogenica* in co-culture with *P. aeruginosa* (Fig. 5C). Together, these observations further supported the reliance of *P. melaninogenica* on mucin for survival and growth in co-culture with *P. aeruginosa*.

### Mucin glycan sugars and amino acids are sufficient to support the growth of *P. melaninogenica* in co-culture with *P. aeruginosa*

**Fig 6:**
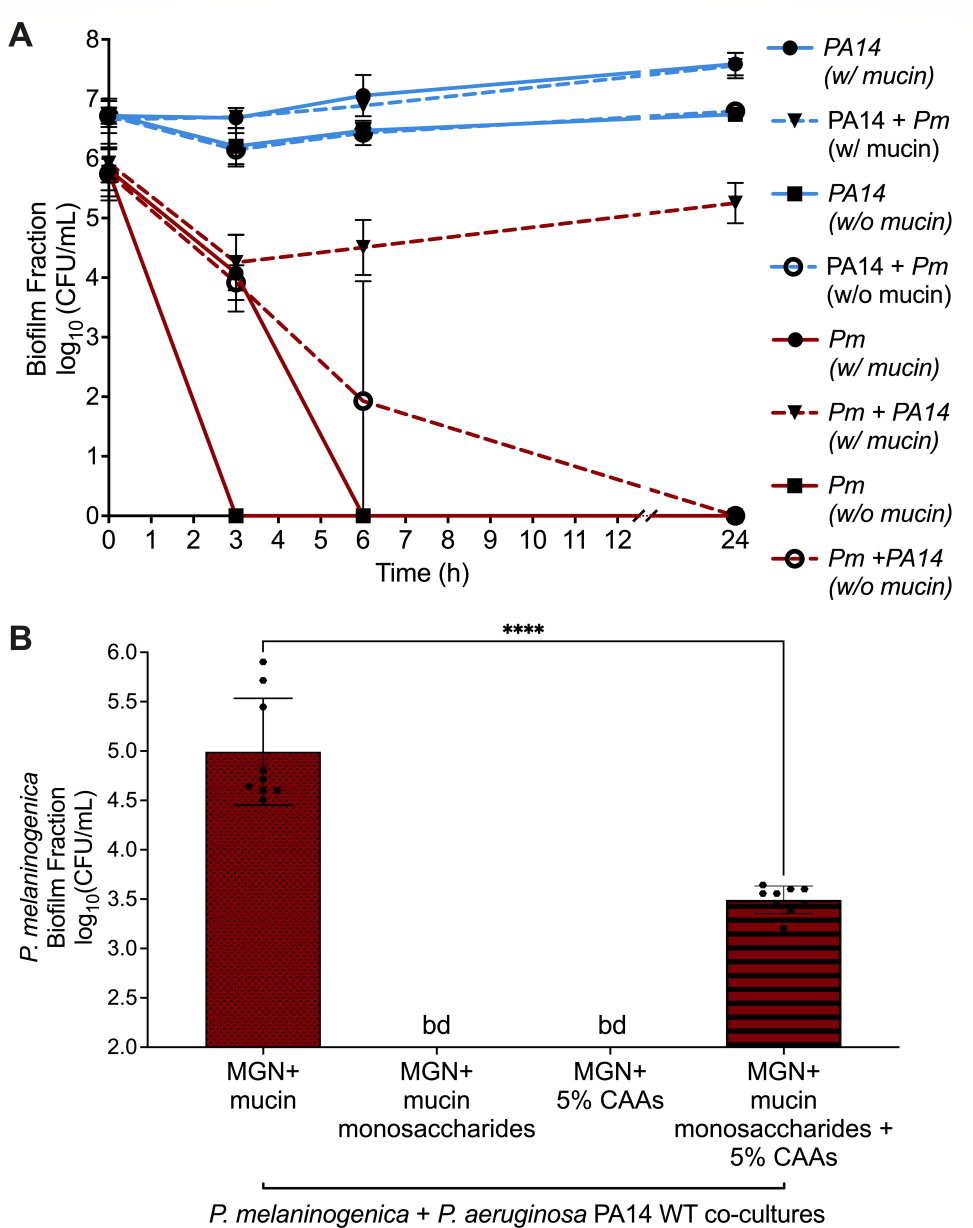
*P. melaninogenica* requires both sugar and amino acid components of mucin to support its growth in co-culture with *P. aeruginosa*. (**A**) Growth in M63 minimal salts supplemented with 0.2% glycerol and 100 µM NO_3_ (called “MGN”) plus 5 mg/mL mucin is sufficient to recapitulate the growth of *P. melaninogenica (Pm*) when co-cultured with *P. aeruginosa (Pa*). The viability of *P. aeruginosa* and *P. melaninogenica* was assessed in mono- and co-cultures by recording the biofilm fraction CFUs at 3, 6, and 24 hours under anoxic growth conditions. The growth of *P. aeruginosa* was unaffected ± mucin in both mono- and co-cultures. *P. melaninogenica* did not survive monoculture conditions beyond the 6 h and 3 h timepoints in minimal medium with and without mucin, respectively. *P. melaninogenica* in co-culture with *P. aeruginosa* and without mucin in the medium experienced a steady decline in viability from ~10^6^ CFU/mL over the 24 h period until it was no longer detectable. Finally, *P. melaninogenica* in co-culture with *P. aeruginosa*, and in the presence of mucin, appeared to require the same 6 h adjustment period observed for growth in mucin-containing ASM (see Fig. 1B) before being able to steadily grow over time to ~10^5^ CFU/mL. (**B**) The growth of *P. melaninogenica* in co-culture with *P. aeruginosa* can be partially supported with a medium containing the mucin components mucin monosaccharides (fucose, galactose, N-acetylglucosamine, and N-acetylgalactosamine) and amino acids (casamino acids, CAA) instead of the crude mucin extract. Statistical significance was calculated using a two-tailed student’s t-test, ****P < 0.0001.

Since ASM is a complex medium composed of amino acids, sugars, mucin, metal ions, and DNA (12) that can complicate the detection of metabolites in the cell-free supernatants, we sought to simplify the co-culture medium by replacing mucin-containing ASM with an M63 minimal salts base medium, supplemented with 0.2% glycerol as an energy source to support *P. aeruginosa* growth, 100 µM nitrate as the anaerobic terminal electron acceptor for *P. aeruginosa*, and mucin at the same concentration used in ASM. The viability of *P. aeruginosa* and *P. melaninogenica* mono- and co-cultures was assayed over time by recording the biofilm fraction, and as shown in Fig. 6A, this medium replicated the growth of *P. melaninogenica* and *P. aeruginosa* in mucin-supplemented ASM, with *P. melaninogenica* only growing in co-culture.

The experiments above indicated that in the context of ASM, added mucin was sufficient for *P. aeruginosa* to support the growth of *P. melaninogenica*. However, the complex and hard-to-characterize nature of mucin complicates our interpretations, prompting us to further simplify the medium to dissect the *P. melaninogenica-P. aeruginosa* interactions. Considering the glycoprotein nature of mucins (37), we investigated the capacity of mucin components (monosaccharides and amino acids) to support the growth of *P. melaninogenica* in co-culture with *P. aeruginosa* in a minimal salts medium with glycerol and nitrate under anoxic growth conditions. The selected mucin glycan sugars were N-acetylgalactosamine, N-acetyl-glucosamine, galactose, and fucose (38), while the amino acid component of mucin (37) was represented by 5% casamino acids (CAAs). The co-culture of *P. melaninogenica* with *P. aeruginosa* in minimal medium containing mucin allowed for the growth of the former to ~10^5^ CFU/ml (Fig. 6B). Interestingly, *P. melaninogenica* was also recoverable, albeit to a significantly lesser amount (3.16 × 10^3^ CFU/mL), when co-cultured with *P. aeruginosa* in minimal medium containing both mucin monosaccharides and CAAs, but not either component individually (Fig. 6B). *P. melaninogenica* was undetectable in all monoculture conditions (data not shown). Here, *P. aeruginosa* was recovered from mono- and co-cultures regardless of whether mucin or its components were present in the minimal medium (Fig. S8). However, *P. aeruginosa* grew to a significantly lesser extent with mucin components compared to mucin in both mono- and co-culture conditions (Fig. S8). Taken together, these data show that a simplified minimal salts medium with mucin components can, at least partially, recapitulate the observed *P. melaninogenica-P. aeruginosa* interaction in ASM + mucin. Furthermore, these data demonstrate that mucin, or carbon compounds derived from mucin, is a key mediator in the interaction between these organisms.

### Metabolic modeling of *P. melaninogenica* on mucin components reveals a metabolic focus on amino acid metabolism and precursors of lipid biosynthesis, including malonate metabolism

In an effort to better understand the metabolic activity of *P. melaninogenica* using mucin, we generated a metabolic model of *P. melaninogenica* grown in a medium composed only of mucin components as a proxy to complete mucin using the ModelSEED/RAST/FBA pipeline that is found on the US Department of Energy (DOE) Systems Biology Knowledgebase (KBase) platform (https://www.kbase.us/) (39–43). The model generally aims to maximize the potential biomass production by an organism with the fewest required metabolic reactions to achieve growth in the given culture condition. The *P. melaninogenica* model unsurprisingly showed very little predicted biomass production from the mucin components, with an Objective Value of just 0.0501 (https://kbase.us/n/223351/72/). Therefore, the predictions of *P. melaninogenica* metabolism of mucin are limited by the available biomass. However, upon performing flux balance analysis (FBA), the model predicted that the pathways with most positive flux in *P. melaninogenica* were those involved in amino acid as well as carbon metabolism. Serine biosynthesis followed by its metabolism into pyruvate was predicted to have a reaction flux of approximately 2.5 in absolute value, among the highest predicted (Table S3, https://kbase.us/n/223351/72/). Furthermore, the model predicted a reaction flux of 2.21 in absolute value for the decarboxylation of pyruvate into acetyl-CoA (Table S3, https://kbase.us/n/223351/72/), which was then predicted to be carboxylated to malonyl-CoA, that is then transferred to an acyl carrier protein to form malonyl-ACP, both of the latter reactions with an approximate reaction flux of 0.11 in absolute value (Table S3, https://kbase.us/n/223351/72/). Malonyl-ACP can then participate in fatty acid biosynthesis (28, 29). Thus, this model does predict the production and utilization of malonate as a part of *P. melaninogenica*’s metabolism.

### *P. melaninogenica* expresses genes implicated in carbon metabolism when grown with *P. aeruginosa* in mucin-containing ASM

**Fig 7:**
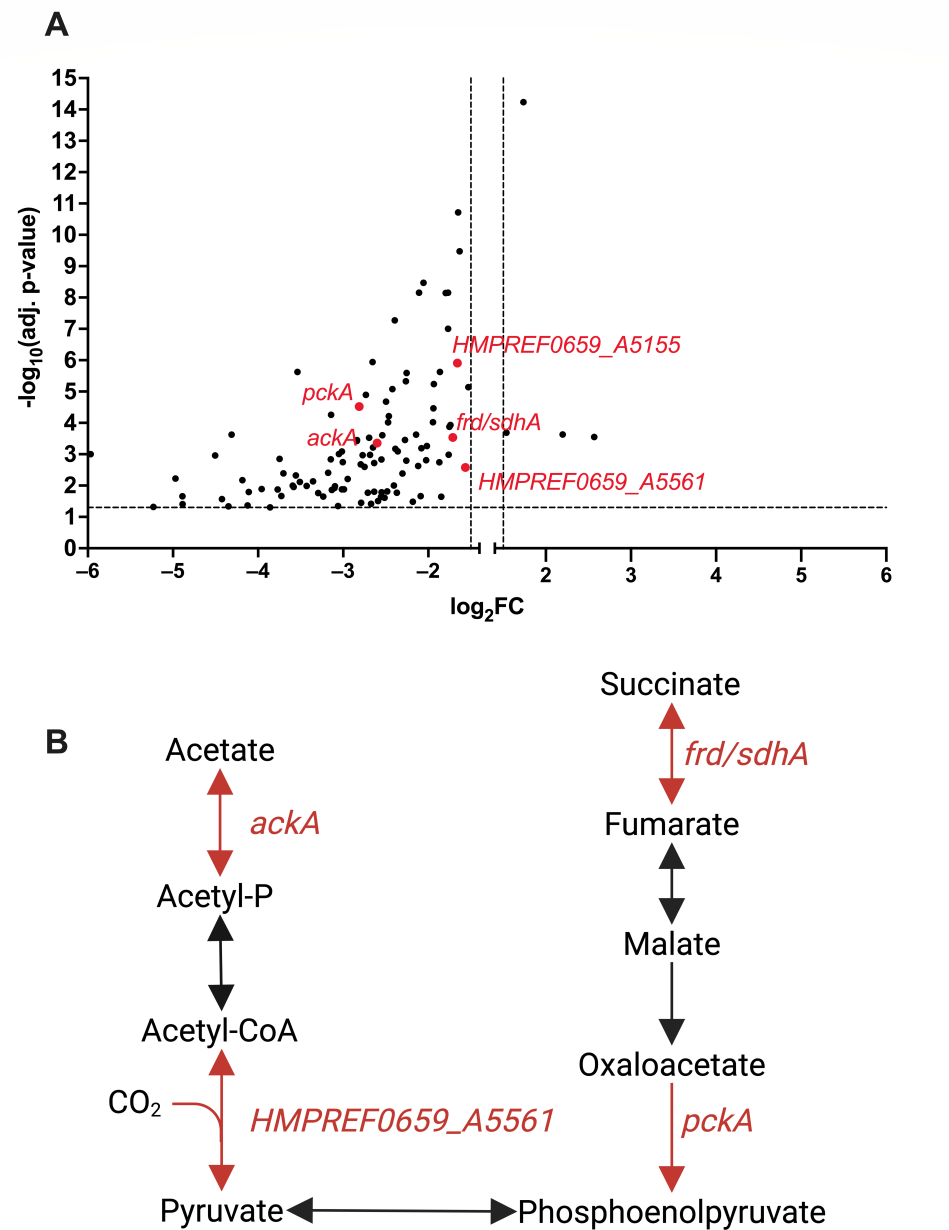
*P. melaninogenica* differentially responds to mucin in the culture medium when co-cultured with the CF polymicrobial community. (**A and B**) The RNAseq data used to generate the figures were originally reported in Kesthely et al. (44) (**A**) A volcano plot depicting the most differentially expressed genes in *P. melaninogenica* upon its co-culture with the CF polymicrobial community model, composed of *P. aeruginosa*, *S. aureus*, *S. sanguinis*, and *P. melaninogenica*, in either mucin-containing ASM (this condition was referred to as “Mix” in the original publication [44]) or ASM lacking mucin (this condition was referred to as “Mix_base” in the original publication (44)). The dots on the left side of the graph indicate genes that are significantly downregulated in the absence of mucin (or upregulated in the presence of mucin), while those on the right side of the graph indicate targets that are significantly upregulated in the absence of mucin (or downregulated in the presence of mucin). (**B**) The *P. melaninogenica* genes highlighted in red are significantly upregulated in the presence of mucin and include genes involved in the metabolism of acetate and succinate.

Previous work from our lab investigated the transcriptional profiles of *P. aeruginosa*, *S. aureus*, *S. sanguinis*, and *P. melaninogenica* as part of the CF-relevant polymicrobial community in ASM with and without mucin (44). Upon visualizing the differential expression data of *P. melaninogenica* when grown as part of the community, it was evident that the presence of mucin was a key factor leading to the differential expression of multiple genes implicated in cellular metabolism, many of which were significantly downregulated in the absence of mucin (Fig. 7A). In fact, upon performing a gene-list enrichment analysis (45) of the most differentially expressed genes in the presence of mucin (Fig. 7A), we found that a number of those genes belonged to pathways involved in the metabolism of acetate and succinate (Fig. 7B), indicating an uptick in carbon metabolism by *P. melaninogenica* in the presence of mucin when part of the polymicrobial community.

**Fig 8:**
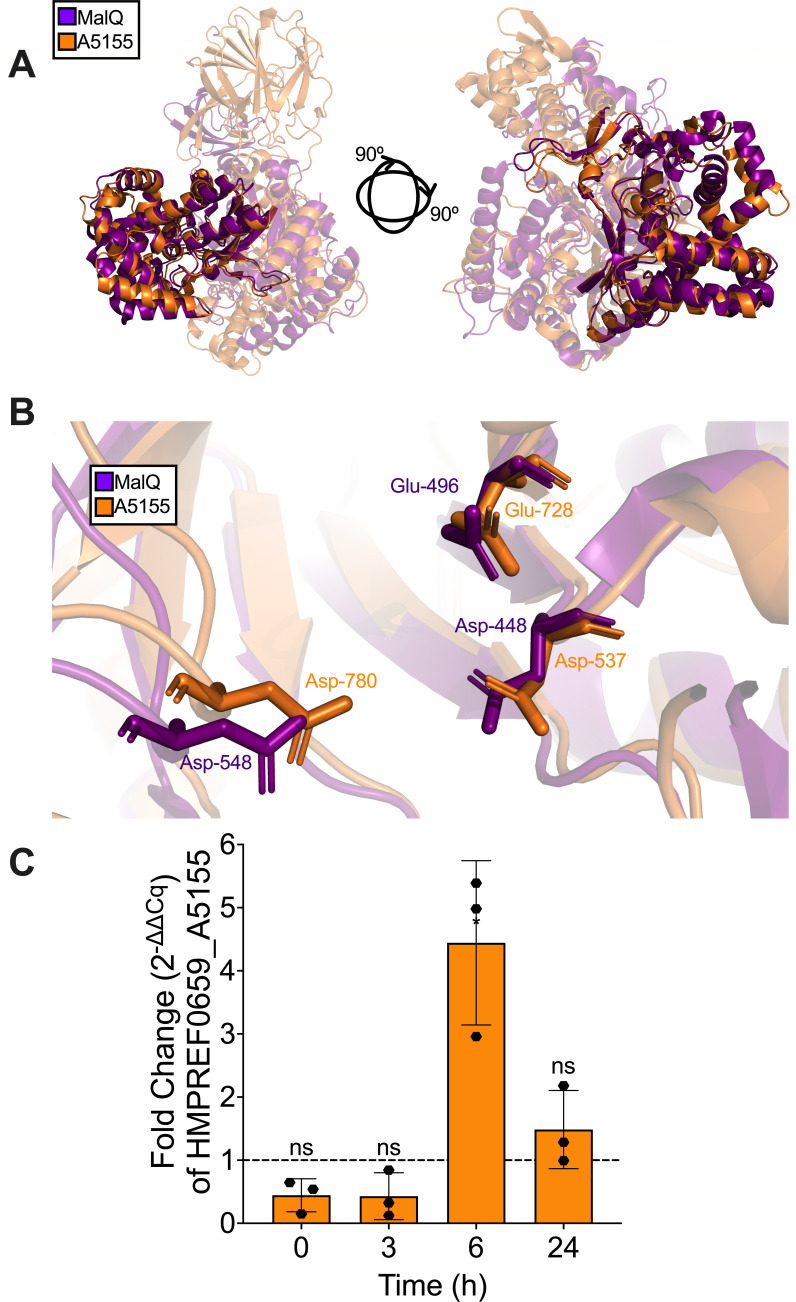
The *P. melaninogenica* CAZyme HMPREF0659_A5155 is upregulated in the presence of mucin during co-culture with *P. aeruginosa*. (**A**) The HMPREF0659_A5155 (A5155) CAZyme is most structurally similar to the 4-α-glucanotransferase enzyme MalQ of *E. coli* K12. (**B**) The *E. coli* MalQ catalytic triad is composed of two Asp and one Glu residues that align with the same residues on A5155. (**C**) There is an approximate 4.5× increase in the fold change of *HMPREF0659_A5155* expression when *P. melaninogenica* is co-cultured with *P. aeruginosa* in mucin-containing ASM at the 6 h time point. *HMPREF0659_A5155* Ct values were normalized to *gyrA* as the reference gene. Statistical significance was calculated using a two-tailed student’s t-test, *P < 0.05.

In addition to the overall increase in metabolism, we identified a particular CAZyme (46) that was significantly increased in expression in *P. melaninogenica* in the presence of mucin. The glycoside hydrolase HMPREF0659_A5155 (A5155) belongs to the glycoside hydrolase 77 (GH77) family (46) and is predicted to be a putative 4-α-glucanotransferase which can transfer a segment of a 1,4-α-D-glucan onto the 4-position of an acceptor molecule (47, 48). To further characterize this protein, we employed a number of computational approaches. First, we analyzed the protein sequence via InterPro (49), which identified a carbohydrate-binding module (CBM) (a.a. 126–233) and a glycoside hydrolase domain (a.a. 248–872). Next, we wanted to identify known proteins that were structurally similar to A5155. We queried the AlphaFold-predicted 3D structure of the protein on the Dali Server (50). The characterized protein with the best Dali Z-score compared to A5155 was MalQ of *E. coli* K12, which encodes a 4-α-glucanotransferase. Although overlaying the two 3D protein structures on PyMOL v3.0 (51) only showed alignment along a portion of the proteins (Fig. 8A), the catalytic triad of MalQ, composed of residues Asp-448, Glu-496, and Asp-548 (52), aligned with residues of the same species, Asp-537, Glu-728, and Asp-780 on A5155 (Fig. 8B). Therefore, we can postulate that the poorly characterized *P. melaninogenica* protein A5155 is functionally similar to MalQ.

To investigate the transcription of this enzyme in *P. melaninogenica*, we grew the organism anaerobically in co-culture with *P. aeruginosa* in mucin-containing ASM and measured the fold change of the expression of the gene over time via RT-qPCR. It was evident that the gene was not expressed in the early stages of the co-culture before displaying a ~4.5 fold increase at the 6 h time point, then returning to around baseline by the end of the co-culture (Fig. 8C). This observation coincides with the growth curve of *P. melaninogenica* in co-culture with *P. aeruginosa* (Fig. 1B) where it begins showing an increase in the mucin-containing ASM starting at the 6 h time point. Therefore, it is possible that *P. melaninogenica* utilizes A5155 to metabolize sugar moieties of the mucin glycoprotein structure, and perhaps contributes to the *P. aeruginosa-P. melaninogenica* interaction.

## DISCUSSION

In this study, we leveraged an existing CF airway polymicrobial community model, consisting of *P. aeruginosa, S. aureus, S. sanguinis,* and *P. melaninogenica* (11) to investigate the mechanisms that govern the ability of *P. melaninogenica* to grow in co-culture with the other members of this CF polymicrobial community, but not as a monoculture in mucin-containing ASM. By co-culturing *P. melaninogenica* in different combinations with other members, we identified *P. aeruginosa* as the main supporter of the survival of *P. melaninogenica* in the polymicrobial community. The inclusion of *P. aeruginosa* in the culture consistently improved the recovery of *P. melaninogenica* irrespective of the presence of *S. aureus* and *S. sanguinis* (Fig. 1A). The growth of *P. aeruginosa* in co-culture did not depend on the presence of *P. melaninogenica*. This observation aligns with our previous transcriptomic study of the same four-membered polymicrobial community, where *P. aeruginosa* showed an agnostic transcriptional response to the other members of the community (44). In addition, *P. aeruginosa* respires anaerobically via denitrification (53–55), and nitrate is provided in the medium. On the other hand, *P. melaninogenica* could not survive monoculture beyond 6 hours and appeared to require this first 6 hours, perhaps in a lag phase, before growing in co-culture with *P. aeruginosa* (Fig. 1B). Initially, the lack of monoculture growth by *P. melaninogenica* might be attributed to the lack of hemin and menadione in ASM, as both are usually required for the cultivation of *Prevotella* spp. *in vitro* (56, 57); however, the addition of both hemin and menadione to ASM + mucin was still insufficient to recover *P. melaninogenica* growth in anoxic monoculture (Fig. 1C). Therefore, we hypothesized that *P. aeruginosa* must be providing certain essential nutrients to support the growth of *P. melaninogenica* in a CF-like environment.

To further elucidate the mechanism that governs the interaction between these organisms, we screened the *P. aeruginosa* PA14 non-redundant transposon mutant library (18) in search of mutants that were incapable of supporting the growth of *P. melaninogenica* to the same extent as wild-type *P. aeruginosa* PA14 in co-culture. We identified carbon and amino acid metabolism in *P. aeruginosa* PA14 as key pathways implicated in the interaction between these organisms (Table S1). Previous metabolic modeling of the CF-relevant polymicrobial community predicted that organic acids were cross-fed between members of the model community (8, 11). While we did not identify mutations in the pathways predicted by this metabolic modeling, we thought that the organic acid-metabolizing pathways identified in our screen were reasonable to pursue here. Therefore, we experimentally interrogated the malonate and propionate metabolism pathways that were identified by the screen (Fig. 2B). To this end, we co-cultured *P. melaninogenica* with *P. aeruginosa* PA14 deletion mutants of the *mdc* and *prp* genes, rendering *P. aeruginosa* incapable of converting malonate to acetate and propionate to succinate, respectively. The viability of *P. melaninogenica* was significantly reduced by ~10-fold when anoxically co-cultured in mucin-containing ASM with any of *P. aeruginosa* PA14 Δ*mdcA,* D*mdcC*, Δ*mdcE,* D*prpB,* and Δ*mdcC*D*prpB* mutants compared to WT *P. aeruginosa* PA14 (Fig. 2C–D). These observations indicated that the disruption of acetate and succinate production in *P. aeruginosa* can significantly handicap the growth of *P. melaninogenica* in co-culture under CF-like conditions. Thus, acetate and succinate, as well as their respective metabolic precursors, malonate and propionate, seem to be potential mediators that are shared between organisms to help *P. melaninogenica* survive the CF polymicrobial environment. In addition, we demonstrated that other metabolic pathways in *P. aeruginosa* that result in the formation of acetate and succinate (Fig. 3A), namely those proteins encoded by the *pauA*, *sucDC*, and *sdhBADC* genes, can also impact the interaction between *P. aeruginosa* and *P. melaninogenica*. Indeed, we observed that *P. melaninogenica* exhibits reduced viability upon its co-culture with those mutants in mucin-containing ASM (Fig. 3B).

Interestingly, supplementing acetate or succinate into the co-culture of *P. melaninogenica* with the *P. aeruginosa* mutants at 6 hours (but not at the start of the experiment) restored the *P. melaninogenica* viable counts to those observed when it was co-cultured with WT *P. aeruginosa* PA14 (Fig. 2C and D, 3B and C). This 6 h time point aligns with the time course co-culture assay results, indicating that the promotion of *P. melaninogenica* growth by *P. aeruginosa* apparently requires a period of adaptation—perhaps this time is needed for the requisite metabolites to be produced by the organisms and for cross-feeding to initiate. We do not understand why supplementing the ∆*sucDC,* ∆*sucDC*∆*prpB*, or ∆*sucDC*∆*mdcC* with succinate did not rescue growth, but we speculate that alterations in succinyl-CoA production might be having unanticipated effects on cell physiology.

We were able to establish that supernatants from co-cultures of *P. melaninogenica-P. aeruginosa*, but not either organism alone, could support the growth of *P. melaninogenica* in monoculture (Fig. 1C). However, the fact that *P. melaninogenica* growth decreased with successive rounds of incubation in the *P. melaninogenica-P. aeruginosa* co-culture supernatant (Fig. 1C) indicated that *P. melaninogenica* was likely consuming the metabolic products found in the supernatants. Thus, it does not appear that *P. aeruginosa* cross-feeding is required to simply “kick start” *P. melaninogenica* growth. In addition, we observed that the co-culture supernatants of *P. aeruginosa* metabolic mutants with *P. melaninogenica* cannot support a *P. melaninogenica* monoculture to the same extent as the WT PA14-*Pm* supernatant (Fig. 3D). Together, our observations demonstrate the reliance of *P. melaninogenica* on the metabolic products produced by *P. aeruginosa* when these two organisms are grown in co-culture. Furthermore, we were able to measure increased concentrations of acetate and succinate specifically in the *P. melaninogenica-P. aeruginosa* co-culture supernatant (Fig. 4A). Consistent with our cross-feeding model, we note that the concentrations of acetate and succinate in the *P. melaninogenica-P. aeruginosa* co-culture supernatant were significantly reduced after culturing of *P. melaninogenica* (Fig. 4B). Together, our data are consistent with a model wherein *P. melaninogenica* and *P. aeruginosa* must be viable and co-cultured for *P. melaninogenica* to grow in the CF airway-like ASM + mucin medium, and that this growth promotion of *P. melaninogenica* is at least in part due to cross-feeding of organic acids.

The detection of acetate and succinate at higher concentrations in the co-cultures of our CF-like *in vitro* model also has physiological relevance. A previous study reported that the median acetate concentration was found to be more than double in the exhaled breath of 58 pwCF at 178 parts-per-billion by volume (ppbv) compared to healthy controls at 80 ppbv (58). Likewise, succinate was determined to be 10-fold higher in the sputa of 23 pwCF compared to 19 healthy controls (59). And third, the concentrations of short-chain fatty acids (SCFAs) in the sputa of 9 pwCF were previously reported to be between 0.82 and 4.06 mM (60), which is near the range of the concentrations we detected in our studies here.

The cross-feeding of malonate, propionate, acetate, and succinate does not fully explain the mechanism underlying the interaction between *P. melaninogenica* and *P. aeruginosa* since the disruption of their metabolic pathways, either individually or in conjunction, did not result in the complete loss of *P. melaninogenica* detection in co-cultures as observed with monocultures. Also, the addition of malonate, propionate, acetate, succinate, and fumarate, the terminal electron acceptor in *P. melaninogenica* anaerobic respiration, did not rescue the growth of *P. melaninogenica* monocultures (Fig. S2C), indicating the involvement of additional metabolic pathways in the interaction process. Therefore, we co-cultured *P. melaninogenica* with *P. aeruginosa* PA14 carbon catabolite repression mutants to evaluate the effect of disrupting broader metabolic pathway regulation on the interaction between the organisms. We observed that *P. aeruginosa* PA14 Δ*cbrA,* Δ*cbrB,* and Δ*crc* mutants supported the growth of *P. melaninogenica* to a significantly lesser extent than WT *P. aeruginosa* PA14 (Fig. S5). Such a finding highlights the complexity of the mechanisms governing the interaction between *P. melaninogenica* and *P. aeruginosa* since CCR is involved in regulating the uptake and metabolism of different carbon sources, amino acids, lipids, and nucleic acids, as well as phenazine biosynthesis and the PQS system (35, 61). Interestingly, some CCR targets include *ascA* and *prpC* (35, 62), which are involved in propionate metabolism, *hutU* (63), which is involved in histidine metabolism, and *dctA*, which is a C_4_-dicarboxylate transporter of succinate, fumarate, and malate (35, 36), highlighting the importance of the ability of *P. aeruginosa* to transport organic acids when cross-feeding *P. melaninogenica*.

We were also able to demonstrate that *P. aeruginosa* does not utilize the mucin found in ASM since its growth as a monoculture was not impacted by the presence of mucin (Fig. 5A). These data align with previously published observations (31). Interestingly, that same publication also demonstrated that a CF-derived consortium of anaerobic bacteria, which includes *P. melaninogenica*, was able to ferment mucin into short-chain fatty acids, including acetate, propionate, and lactate (31).

**Fig 9:**
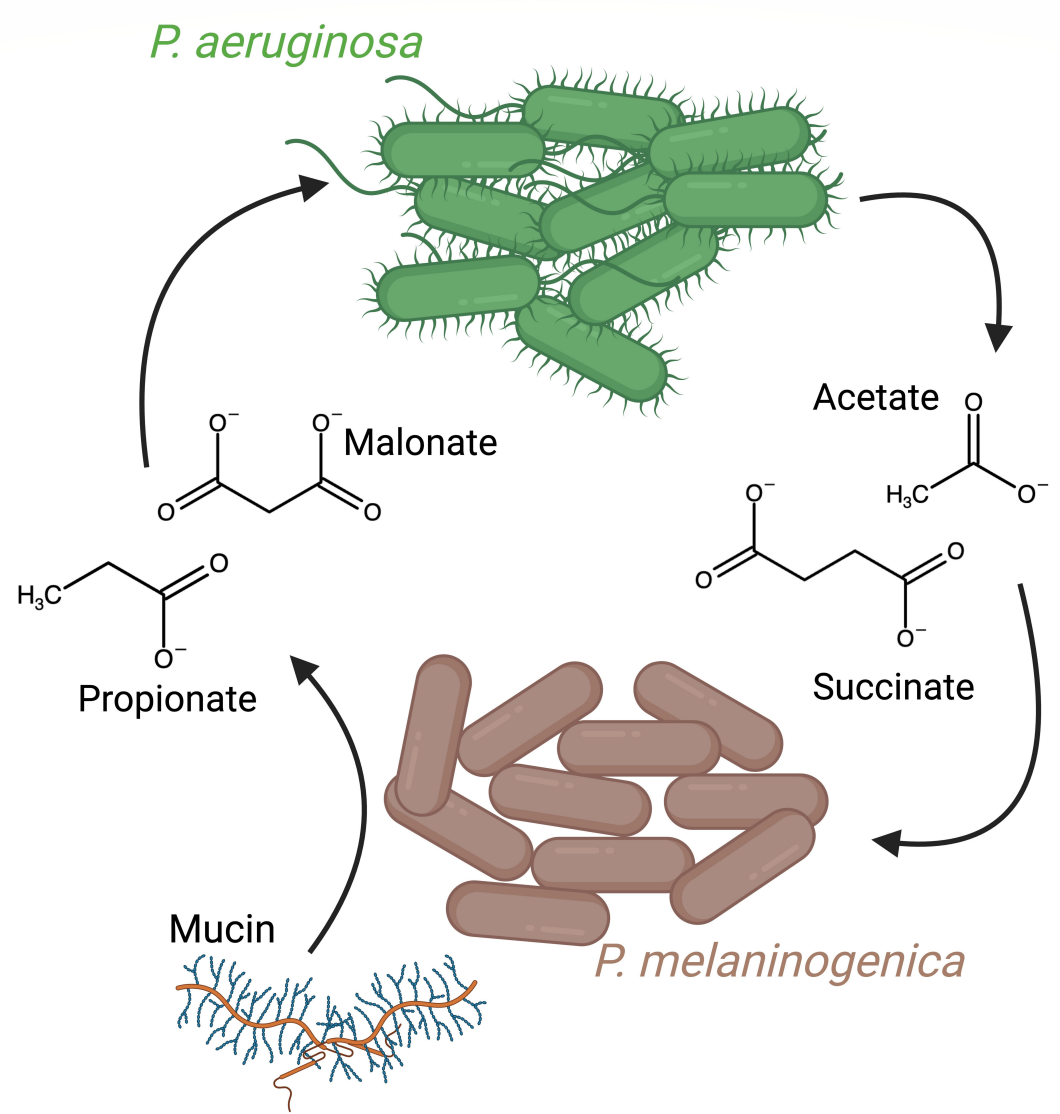
A proposed *P. aeruginosa-P. melaninogenica* interaction model. In this model, based in part on the data presented here, *P. melaninogenica* metabolizes mucin into malonate and propionate, which are then metabolized by *P. aeruginosa* into acetate and succinate, respectively. Acetate and succinate then serve to promote the growth of *P. melaninogenica* in this CF lung-like environment. The metabolic cross-feeding illustrated here likely represents only a subset of the possible interactions but is based on the genetic, modeling, and targeted metabolomics data presented here. Figure designed using BioRender.

By coupling these observations with experimental data demonstrating the dependency of *P. melaninogenica* on mucin in co-culture with *P. aeruginosa* (Fig. 5B and C, Fig. 6) and prior transcriptomic data showing the reliance of *P. melaninogenica* on mucin in ASM (44), along with RT-qPCR data that indicate the capability of *P. melaninogenica* to induce the expression of CAZymes potentially implicated in mucin metabolism (Fig. 7), as well as our data suggesting that only the *P. aeruginosa-P. melaninogenica* co-culture supernatants, not monoculture supernatants, can sustainably support *P. melaninogenica* monocultures (Fig. 1C), we propose a model of interaction between *P. aeruginosa* and *P. melaninogenica* in our CF-relevant community that relies on a two-way cross-feeding mechanism. We posit that *P. melaninogenica* ferments mucin into simpler carbon sources, such as malonate and propionate, during the first 6 hours of the co-culture. Our metabolic modeling here and previous published reports (31, 64–67) support the ability of *P. melaninogenica* to produce malonate and propionate, respectively. Next, we propose that *P. aeruginosa* metabolizes malonate and propionate into acetate and succinate, respectively, and cross-feeds these metabolites back to *P. melaninogenica* to support its growth in a CF-like environment (Fig. 9). As mentioned above, this model does not encompass the entire mechanism of interaction between the two organisms but presents testable hypotheses that can be probed further in future studies.

## MATERIALS AND METHODS

### Bacterial strains and culture conditions

*P. aeruginosa* PA14 (68), *S. aureus* Newman (69), *S. sanguinis* SK36 (70), and *P. melaninogenica* ATCC 25845 (71) were used in this study and cultured in accordance with previously described methods (11). Briefly, *P. aeruginosa* and *S. aureus* were grown overnight in LB (lysogeny broth) at 37°C with shaking. *S. sanguinis* was grown overnight in Todd-Hewitt broth with 0.5% yeast extract at 37°C with 5% CO_2_. *P. melaninogenica* was grown anoxically overnight at 37°C in modified tryptic soy broth yeast extract (TSBYE), composed of tryptic soy broth (TSB) with 0.5% yeast extract, 5 µg/mL hemin, 500 µg/mL L-cysteine, and 1 µg/mL menadione. The list of strains used in this study is found in Table S2.

### Bacterial co-culture assays

All co-culture assays conducted in this study followed a procedure similar to what was previously published (11), with specific adjustments made to suit each experiment as detailed in the text. Generally, assays were performed in 96-well plates. Overnight liquid cultures of the test bacterial strains were collected, pelleted, and washed twice with 1× PBS, except for *P. melaninogenica* and *S. sanguinis*, which were washed once. Afterward, the optical density (OD_600_) of the cultures was normalized to 0.05 in either mucin-containing ASM or minimal medium. The OD-normalized cultures were then either dispensed into the 96-well plate for a final OD_600_ = 0.01 or mixed so that each member would have a final OD_600_ = 0.01, and then the mixture was dispensed into the 96-well plate. Plates were then enclosed in Thermo Fisher Scientific AnaeroPack anaerobic boxes along with a BD GasPak anaerobe sachet and incubated at 37°C for 24 hours. Following incubation, the planktonic fractions of the cultures were separated from the biofilm fraction in the 96-well plate, and 50 µL of 1× PBS was added to each test well, and the biofilm was mechanically detached using a 96-pin replicator. The detached biofilm fractions then underwent 10-fold serial dilutions, and the entire dilution series was spotted onto selective media. CFUs were enumerated following overnight incubation, and the CFU per milliliter concentrations were determined.

The selective media used in the co-culture assays were as follows: *Pseudomonas* isolation agar (PIA) was used to recover *P. aeruginosa*, mannitol salt agar (MSA) was used to recover *S. aureus*, *Streptococcus* selective agar (SSA), made of blood agar supplemented with 10 µg/mL polymyxin B and 10 µg/mL oxolinic acid, was used to recover *S. sanguinis*, and *Prevotella* selective agar (PSA), composed of blood agar supplemented with 5 µg/mL hemin, 500 µg/mL L-cysteine, 1 µg/mL menadione, 100 µg/mL kanamycin, 7.5 µg/mL vancomycin, and 5 µg/mL polymyxin B, was used to recover *P. melaninogenica*. Cultures were incubated for 24 hours unless otherwise noted.

When co-culture supplementation experiments were performed with malonate, propionate, acetate, succinate, and fumarate, 100 mM stock solutions were prepared and then diluted in mucin-containing ASM before being introduced into the cultures at final concentrations of 5, 5, 4.5, 2.8, and 5 mM, respectively, at the 6 h time point.

### Time course co-culture assays

The time-course co-culture assays used in this study relied on the same experimental procedure as the endpoint co-culture assays described above; however, multiple plates in anaerobic boxes were inoculated in parallel, starting from the same overnight cultures, with each anaerobic box corresponding to a timepoint at which the cultures were processed as described above.

### *P. aeruginosa-P. melaninogenica* co-culture genetic screen

The transposon mutagenesis screen utilized the *P. aeruginosa* PA14 non-redundant transposon (Tn) mutant library (18) and was adapted from a previously described procedure (72) with modifications to accommodate *P. melaninogenica*. On the first day, the PA14 Tn library mutants were transferred into a 96-well plate containing 100 µL of LB broth and incubated overnight at 37°C. In parallel, modified TSBYE liquid cultures of *P. melaninogenica* were started and incubated anoxically overnight at 37°C. On the second day, the *P. melaninogenica* cultures were OD-adjusted in mucin-containing ASM to an OD_600_ = 0.01, then dispensed into a 96-well plate. To that same plate, the PA14 Tn library grown overnight in LB was transferred using a 96-pin replicator. Thus, each well contained WT *P. melaninogenica* and a Tn mutant of *P. aeruginosa* PA14. The co-cultures were then incubated anoxically for 24 hours at 37°C. Following incubation, the planktonic fractions of the co-cultures were separated from the biofilm fractions, and 50 µL of 1× PBS was added to the co-culture plates. The biofilm fractions were then mechanically detached using a 96-pin replicator, and each co-culture plate was spotted onto PIA and PSA plates. The PIA plate was then incubated aerobically, and the PSA plate was incubated anoxically overnight at 37°C. Following incubation, *P. aeruginosa* Tn mutants that were recovered on the PIA plate but were unable to (or showed reduced ability to) support the growth of *P. melaninogenica* were identified. Candidate *P. aeruginosa* Tn mutants were individually retrieved from the transposon library and transferred to a separate 96-well plate to generate the primary candidate library. Since the phenotype observed relies on microbial interactions, which are influenced by bacterial cell densities, and the high-throughput setup of a genetic screen does not control for variations in bacterial cell densities between the *P. aeruginosa* Tn mutants and *P. melaninogenica*, a second round of screening was performed using the same procedure, but with strains selected from the primary candidate library, followed by a third round to generate the final list of *P. aeruginosa* PA14 Tn mutants that were unable to effectively support the growth of *P. melaninogenica* in co-culture (Table S1). The selected mutants from the final list were then subjected to confirmatory *P. aeruginosa-P. melaninogenica* co-culture experiments as described in the previous section to verify the phenotypes observed in the genetic screen.

### *P. aeruginosa* PA14 gene deletions

The clean deletion mutants of *P. aeruginosa* PA14 *∆mdcA*, *∆mdcC,* and *∆mdcE*, as well as the complementation mutant PA14 *∆mdcC::mdcC,* were acquired from the Dietrich Lab (25). The clean deletion mutants *P. aeruginosa* PA14 Δ*prpB*, Δ*acsA*, and Δ*prpB*D*acsA* were acquired from the Hunter Lab (31). The *P. aeruginosa* PA14 *∆prpB∆mdcC* mutant was generated in the PA14 *∆mdcC* background via conjugation with *E. coli* S17-1 harboring the deletion vector pSMV8::*prpB*-KO provided by the Hunter Lab (31). The *P. aeruginosa* PA14 ∆*pauA* and ∆*sucDC* were generated via conjugation with *E. coli* S17-1 harboring either pMQ30::*pauA*-KO, pEX18Gm::*sucDC*-KO, or pEX18Gm::*sdhBADC*-KO made in-house. The combination mutants were created by conjugating different *P. aeruginosa* PA14 mutants with *E. coli* S17-1 harboring the desired deletion vector. The clean deletion mutants *P. aeruginosa* PA14 *∆cbrA*, *∆cbrB,* and *∆crc* were acquired from the Hogan Lab (34) along with PA14 ∆*mvfR*/*pqsR* and ∆*pqsH*. The clean deletion mutants *P. aeruginosa* PA14 ∆*pvdA,* ∆*pchE,* ∆*pvdA*∆*pchE,* and ∆*phzA1*∆*phzA2* were acquired from the Newman Lab (73). The list of strains used in this study is found in Table S2.

### Metabolite quantification

Supernatants resulting from the *P. aeruginosa-P. melaninogenica* co-cultures grown in mucin-containing ASM were collected, centrifuged, sterilized through a 0.22 µm filter, and frozen at −80°C in 1.5 mL microcentrifuge tubes. The samples were then shipped to the Mass Spectrometry and Metabolomics Core (MSMC) at Michigan State University, where they were analyzed via gas chromatography-mass spectrometry (GC-MS) using protocols MSU_MSMC_010 and MSU_MSMC_010a (https://rtsf.natsci.msu.edu/mass-spectrometry/protocols.aspx). The organic acid concentrations were calculated by normalizing their values to standards, then normalizing those to blank ASM as a baseline.

### *P. melaninogenica* transcriptional profile analysis

The transcriptional profile of *P. melaninogenica* in the presence and absence of mucin as part of the CF-relevant polymicrobial community was previously investigated (44). Table S4 in this previous publication (44) contains the data required for the analysis, which was retrieved, and the *P. melaninogenica* genes with a log_2_ fold change greater than the absolute value of 1.5 were plotted against the -log_10_ of the adjusted *P*-values in a volcano plot format using GraphPad Prism. As for gene-list enrichment analysis, the KOBAS-i (http://bioinfo.org/kobas/) tool (45) was utilized to identify the KEGG pathways that were significantly enriched in *P. melaninogenica* in the presence of mucin.

### Quantitative reverse transcription polymerase chain reaction

Two separate *P. aeruginosa-P. melaninogenica* co-culture conditions were established as described above. One condition utilized mucin-containing ASM, while the other utilized Modified TSBYE as culture medium. At the 24 h time point, the QIAGEN RNeasy Mini Kit was used in accordance with manufacturer instructions to extract total RNA from the cells of both co-culture conditions. RT-qPCR was run using the following *P. melaninogenica*-specific primers: BH_rt_Pm_A5155_F: 5′-TAGGGTCAGCCAAACGCAAT-3′ and BH_rt_Pm_A5155_R: 5′-TTACATCGTGGTGGTCCTGC-3′ to target the CAZyme HMPREF0659_A5155, and BH_rt_Pm_gyrA_F: 5′-TTACACCGGGTACGTCAAGC-3′ and BH_rt_Pm_gyrA_R: 5′-GACACCGTGAGGAACTCTGG-3′ to target *gyrA* as a reference gene. The Livak (2^−∆∆CT^) method (74) was used to calculate fold change in gene expression with the expression of the CAZyme in the Modified TSBYE co-culture condition acting as the control.

### Metabolic modeling of *P. melaninogenica* grown in mucin

A metabolic modeling analysis of *P. melaninogenica* grown in mucin components was conducted using the US Department of Energy (DOE) Systems Biology Knowledgebase (KBase) platform (https://www.kbase.us/), specifically utilizing the Build Metabolic Model and Run Flux Balance Analysis applications on the platform (39). A genome-scale metabolic model (GEM) of *P. melaninogenica* was first constructed using the ModelSEED2 pipeline (40). The genome was then annotated with the RAST subsystem ontology (42) to generate draft metabolic models incorporating gene-protein-reaction (GPR) associations and biomass reactions tailored to *Prevotella*-specific metabolic capabilities. Media gap-filling was applied (41) to maximize the potential *P. melaninogenica* biomass production in a medium composed only of mucin components as a proxy to complete mucin. To simulate the potential metabolic behavior of *P. melaninogenica*, a flux balance analysis (FBA) computes flux distributions through the *P. melaniniogenica* metabolic network was then performed (43). The metabolic model and flux balance analysis can be found at the following link: https://kbase.us/n/223351/72/. The raw data generated are attached in Table S3.

### Statistical analysis

Analysis was performed using GraphPad Prism 10. The mean values ± standard deviations (SDs) were plotted. Either ordinary one-way analysis of variance (ANOVA) or student’s t-test was performed to determine statistical significance, as indicated in the figure legends.

## SUPPLEMENTAL MATERIAL

Supplemental Figures mbio.01594-25-s0001.pdf Figures S1 to S8.

Table S1 mbio.01594-25-s0002.pdf *P. aeruginosa* PA14 genes identified in the transposon mutagenesis screen.

Table S2 mbio.01594-25-s0003.pdf Strains and plasmids.

Table S3 mbio.01594-25-s0004.xlsx Metabolic model of *P. melaninogenica* on mucin components.

ASM does not own the copyrights to Supplemental Material that may be linked to, or accessed through, an article. The authors have granted ASM a non-exclusive, world-wide license to publish the Supplemental Material files. Please contact the corresponding author directly for reuse.
