# Supplementary material for: Pseudomonas aeruginosa supports the survival of Prevotella melaninogenica in a cystic fibrosis lung polymicrobial community through metabolic cross-feeding
Source: mBio. 2025 Sep 12;16(10):e01594-25. doi: 10.1128/mbio.01594-25 (PMC12506151; doi:10.1128/mbio.01594-25)
Supplement: Supplemental Figures — Figures S1 to S8. [file mbio.01594-25-s0001.pdf]

Supplemental Figures

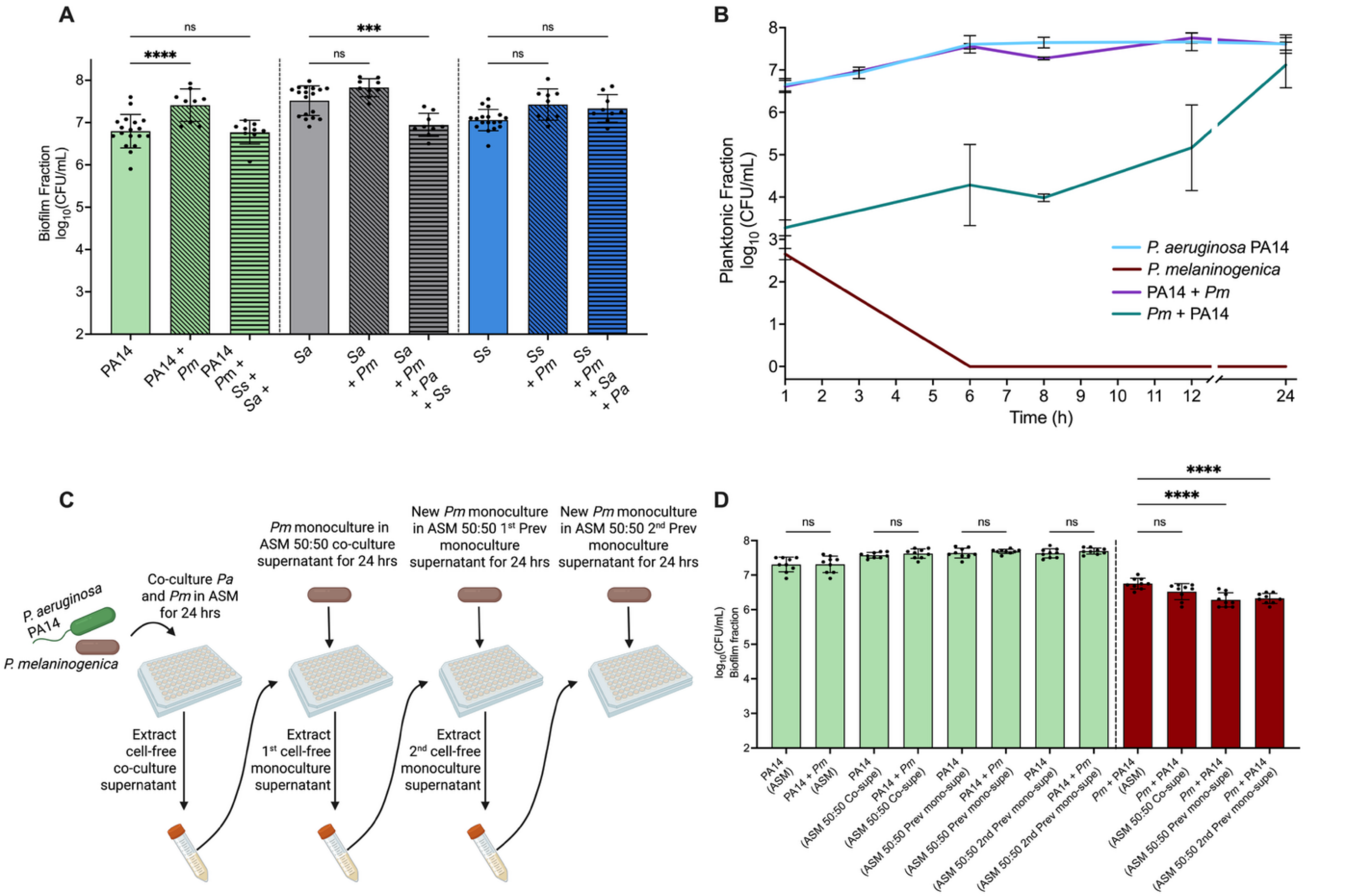

Figure S1. **Viable CFU counts of different polymicrobial communities.** All culture assays were performed using mucin-containing ASM under anaerobic growth conditions at 37ºC for 24 hours. The biofilm fractions of the co-cultures are plotted, unless indicated otherwise. **A.** Viable biofilm counts of *P. aeruginosa* PA14, *S. sanguinis* SK36 (*Ss*) and *S. aureus* Newman (*Sa*) in different co-culture combinations with each other and with *P. melaninogenica* ATCC 25845 (*Pm*). **** $p < 0.0001$, *** $p < 0.005$. **B.** A growth curve of *P. aeruginosa* PA14 and *P. melaninogenica* (*Pm*) CFUs in the planktonic fraction for the mono- and co-cultures using mucin-containing ASM under anaerobic growth conditions at 37ºC. **C.** The experimental setup used to test the effect of cell-free supernatants of different culture conditions on *P. melaninogenica* (*Pm*) monoculture. *P. aeruginosa* PA14 and Pm are co-cultured anoxically in ASM for 24 hrs. The cell-free supernatant that is extracted from the co-culture is then used in a 50:50 ratio with fresh ASM to grow new *Pm* monocultures anoxically for 24 hrs. Finally, the cell-free supernatant of the latter monoculture is then used at a 50:50 ratio with fresh ASM to grow new *Pm* monocultures anoxically for 24 hrs. Figure was created using BioRender **D.** Viable biofilm counts of the control conditions in the experimental setup described in panel C. *P. aeruginosa* PA14 counts as a monoculture and in co-culture with *Pm*, in addition to *Pm* counts in co-culture with PA14. **** $p < 0.0001$. Statistical significance was calculated using ordinary one-way analysis of variance (ANOVA) with Tukey's multiple comparisons test in all panels.

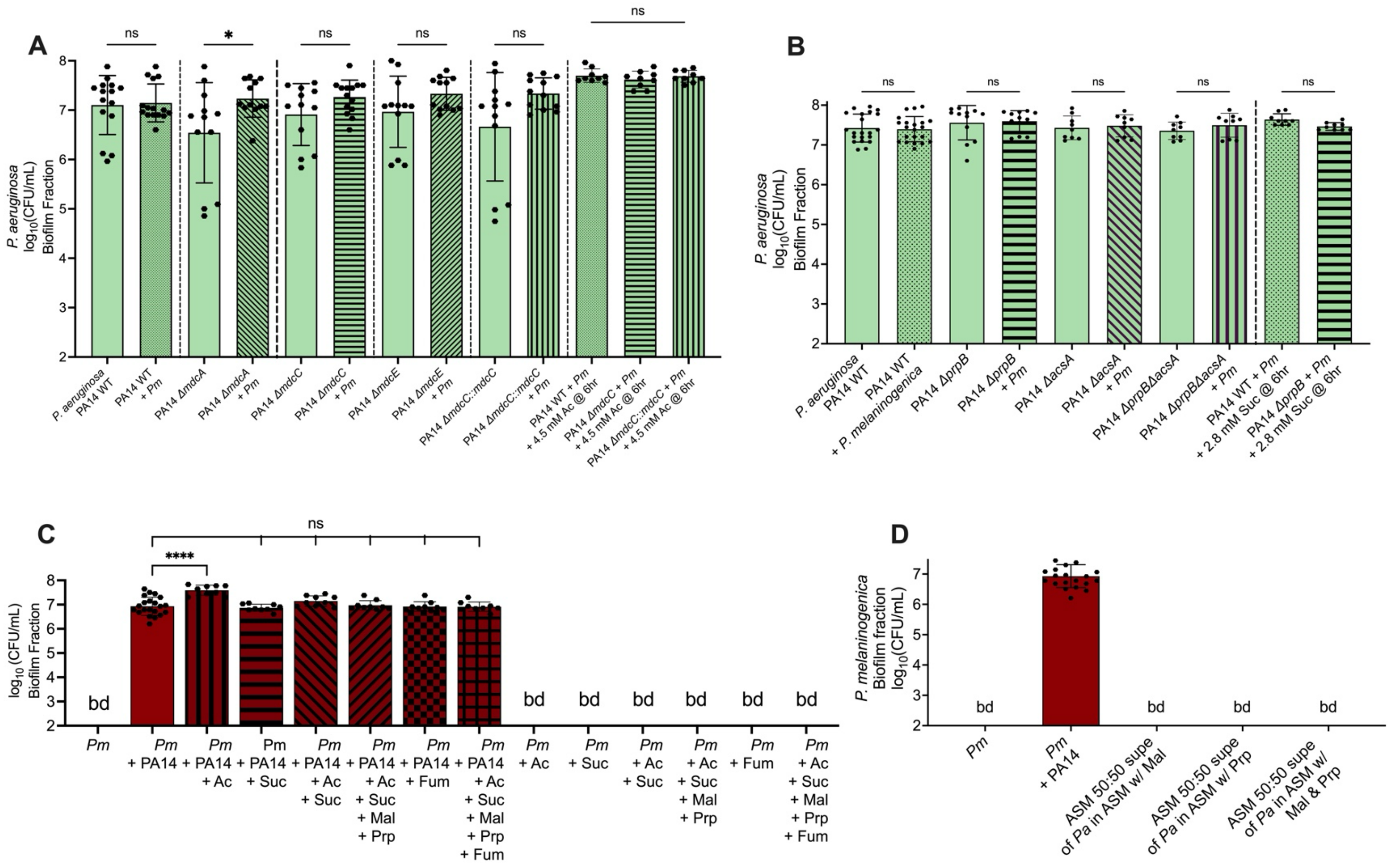

Figure S2. **Wildtype *P. aeruginosa* PA14 and deletion mutants in co-culture with *P. melaninogenica***. **A-C.** All cultures were performed using mucin-containing ASM under anaerobic growth conditions at 37ºC for 24 hours. The biofilm fractions of the co-cultures are plotted. Statistical significance was calculated using ordinary one-way analysis of variance (ANOVA) with Tukey's multiple comparisons. **A.** The mono and co-cultures of WT *P. aeruginosa* PA14 and the *P. aeruginosa* PA14 Δ*mdcA*, Δ*mdcC*, Δ*mdcE* mutants, and the *P. aeruginosa* PA14 Δ*mdcC::mdcC* complement with *P. melaninogenica* (*Pm*) with and without the supplementation of 4.5 mM acetate (Ac) at the 6-hr time point. * $p < 0.05$. **B.** The mono- and co-cultures of WT *P. aeruginosa* PA14 and the Δ*prpB,* Δ*acsA,* and Δ*prpB*Δ*acsA* mutants with *P. melaninogenica* (*Pm*) with and without the supplementation of 2.8 mM succinate (Suc) at the 6-hr time point. **C.** *P. melaninogenica* (*Pm*) mono- and co-cultures with WT *P. aeruginosa* PA14 +/- 4.5 mM acetate (Ac), 2.8 mM succinate (Suc), 5 mM malonate (Mal), 5 mM propionate (Prp), and 5 mM fumarate (Fum). **** $p < 0.0001$. test in all panels. **D.** The monocultures of *P. melaninogenica* in cell-free spent supernatants of WT *P. aeruginosa* PA14 monocultures that were grown in ASM + mucin with malonate (Mal) and/or propionate (Prp), compared to the co-culture of *Pa* and *Pm*.

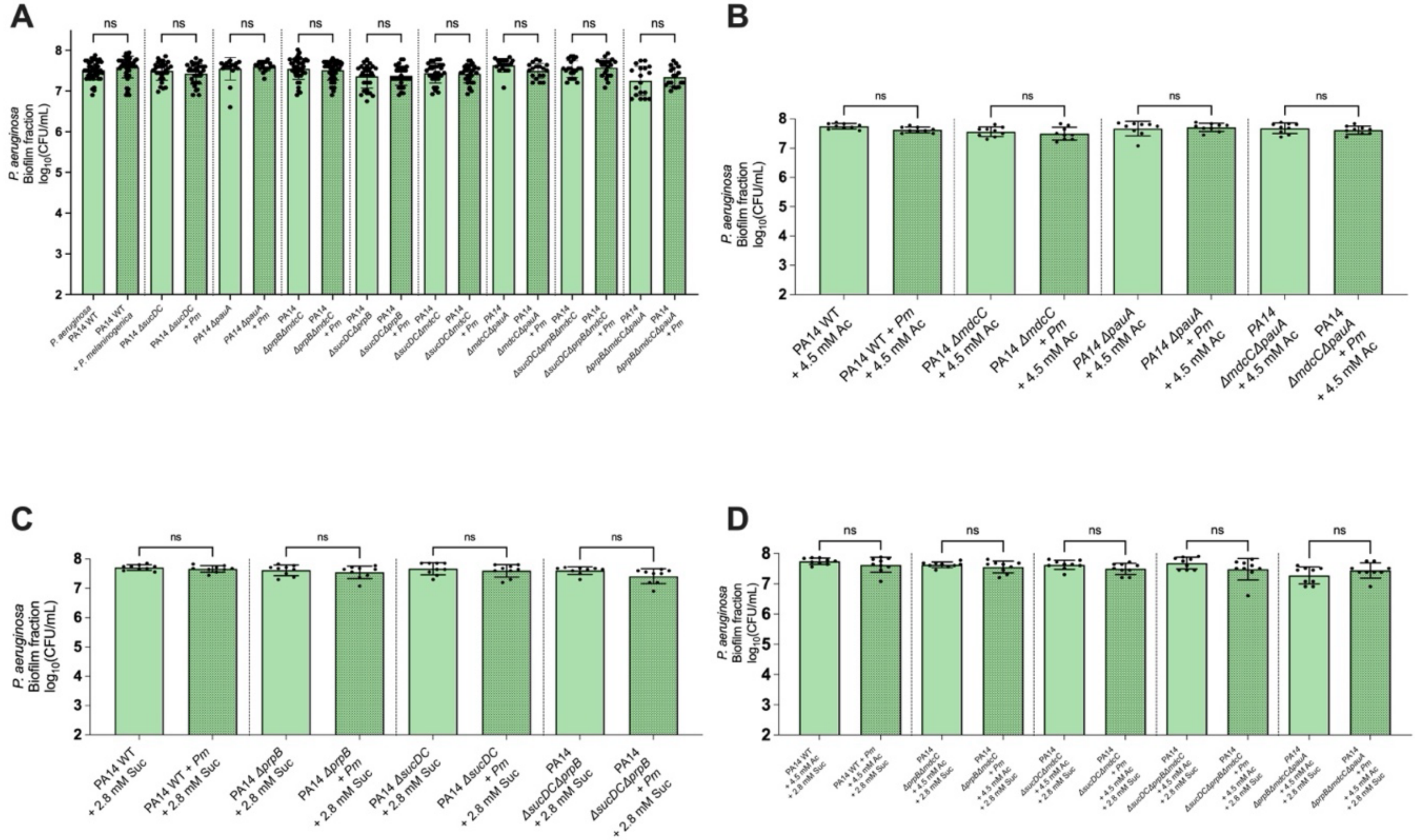

Figure S3. **WT *P. aeruginosa* and the additional *P. aeruginosa* metabolism deletion mutants in co-culture with *P. melaninogenica.* A-D.** All cultures were performed using mucin-containing ASM under anaerobic growth conditions at 37ºC for 24 hours. The biofilm fractions of the co-cultures are plotted. Statistical significance was calculated using ordinary one-way analysis of variance (ANOVA) with Tukey's multiple comparisons test in all panels. **A.** The mono- and co-cultures of WT *P. aeruginosa* PA14 or the Δ*sucDC*, Δ*pauA*, Δ*prpB*Δ*mdcC*, Δ*sucDC*Δ*prpB,* Δ*sucDC*Δ*mdcC*, Δ*mdcC*Δ*pauA*, Δ*sucDC*Δ*prpB*Δ*mdcC*, Δ*sucDC*Δ*prpB*Δ*pauA*, and Δ*sucDC*Δ*prpB*Δ*sdhBADC* mutants with *P. melaninogenica (Pm)*. **B.** The mono- and co-cultures of WT *P. aeruginosa* PA14 or the Δ*mdcC,* Δ*pauA*, and Δ*mdcC*Δ*pauA* mutants with *P. melaninogenica (Pm)* supplemented with 4.5 mM of acetate at the 6-hr time point. **C.** The mono- and co-cultures of WT *P. aeruginosa* PA14 or the Δ*prpB*, Δ*sucDC*, Δ*sucDC*Δ*prpB* mutants with *P. melaninogenica (Pm)* supplemented with 2.8 mM of succinate at the 6-hr time point. **D.** The mono- and co-cultures of WT *P. aeruginosa* PA14 or the Δ*prpB*Δ*mdcC,* Δ*sucDC*Δ*mdcC*, Δ*sucDC*Δ*prpB*Δ*mdcC*, and Δ*sucDC*Δ*prpB*Δ*pauA* mutants with *P. melaninogenica (Pm)* supplemented with 4.5 mM of acetate and 2.8 mM of succinate at the 6-hr time point.

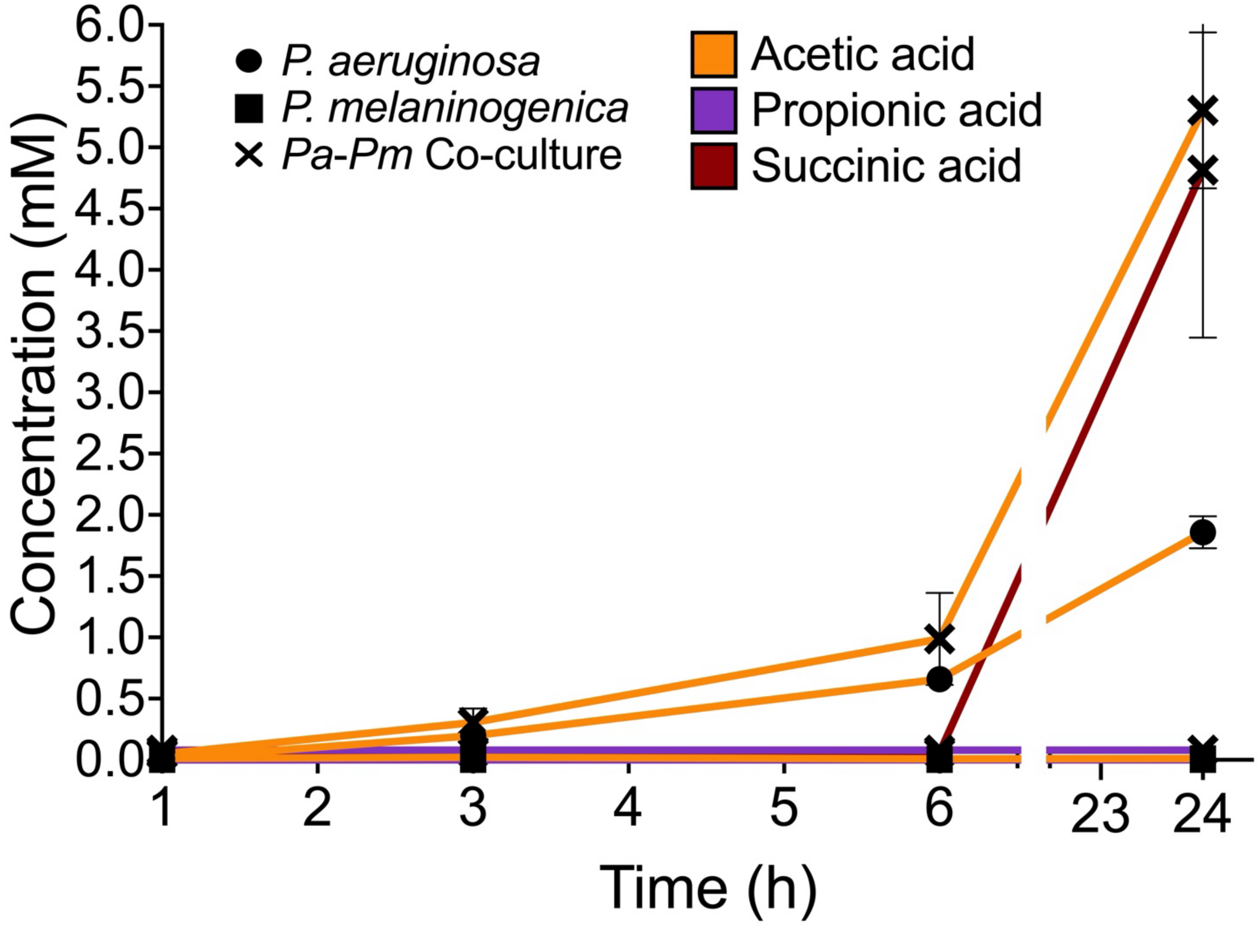

Figure S4. Acetate and succinate concentrations increase over time in co-culture. Plot show levels of the indicated metabolite in mono- versus co-culture at 24 hrs. See Materials and Methods for experimental details. Note that these metabolites do not start appreciably accumulating until after 6 hrs, the time we see *Prevotella* start to grow in co-culture (see **Fig. 1B**).

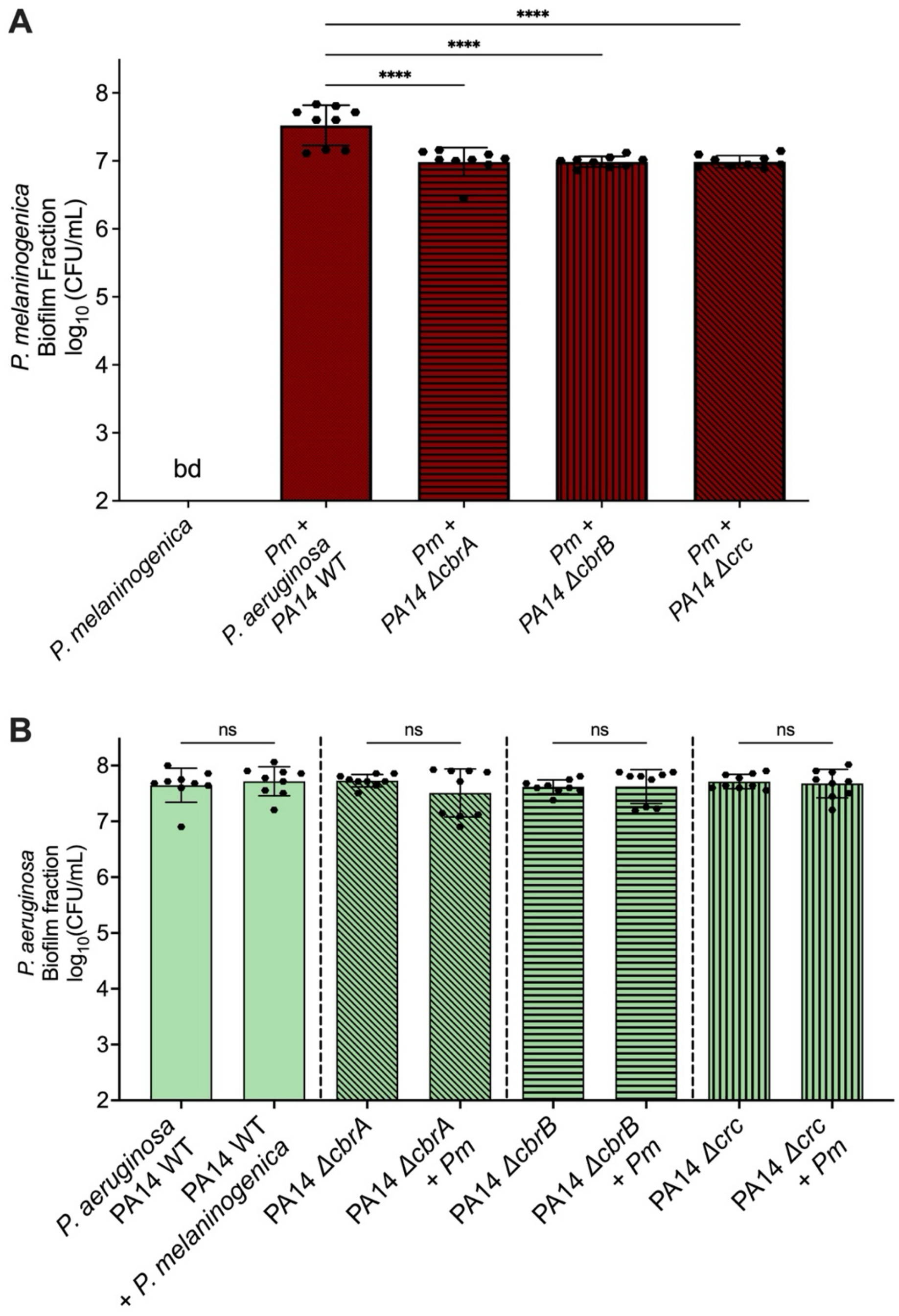

Figure S5. The co-culture of *P. melaninogenica* and *P. aeruginosa* PA14 carbon catabolite repression (CCR) clean deletion mutants. A-B. All cultures were performed using mucin-containing ASM under anaerobic growth conditions at 37ºC for 24 hours. The biofilm fractions of the co-cultures are plotted. Statistical significance was calculated using ordinary one-way analysis of variance (ANOVA) with Tukey's multiple comparisons test for both panels. **A.** The pairwise co-culture of *P. melaninogenica* with WT *P. aeruginosa* PA14 and the *ΔcbrA*, *ΔcbrB*, and *Δcrc* mutants. **** $p < 0.0001$. **B.** The mono- and co-cultures of WT *P. aeruginosa* PA14 and the ∆*cbrA,* ∆*cbrB, and* ∆*crc* mutants with *P. melaninogenica (Pm).*

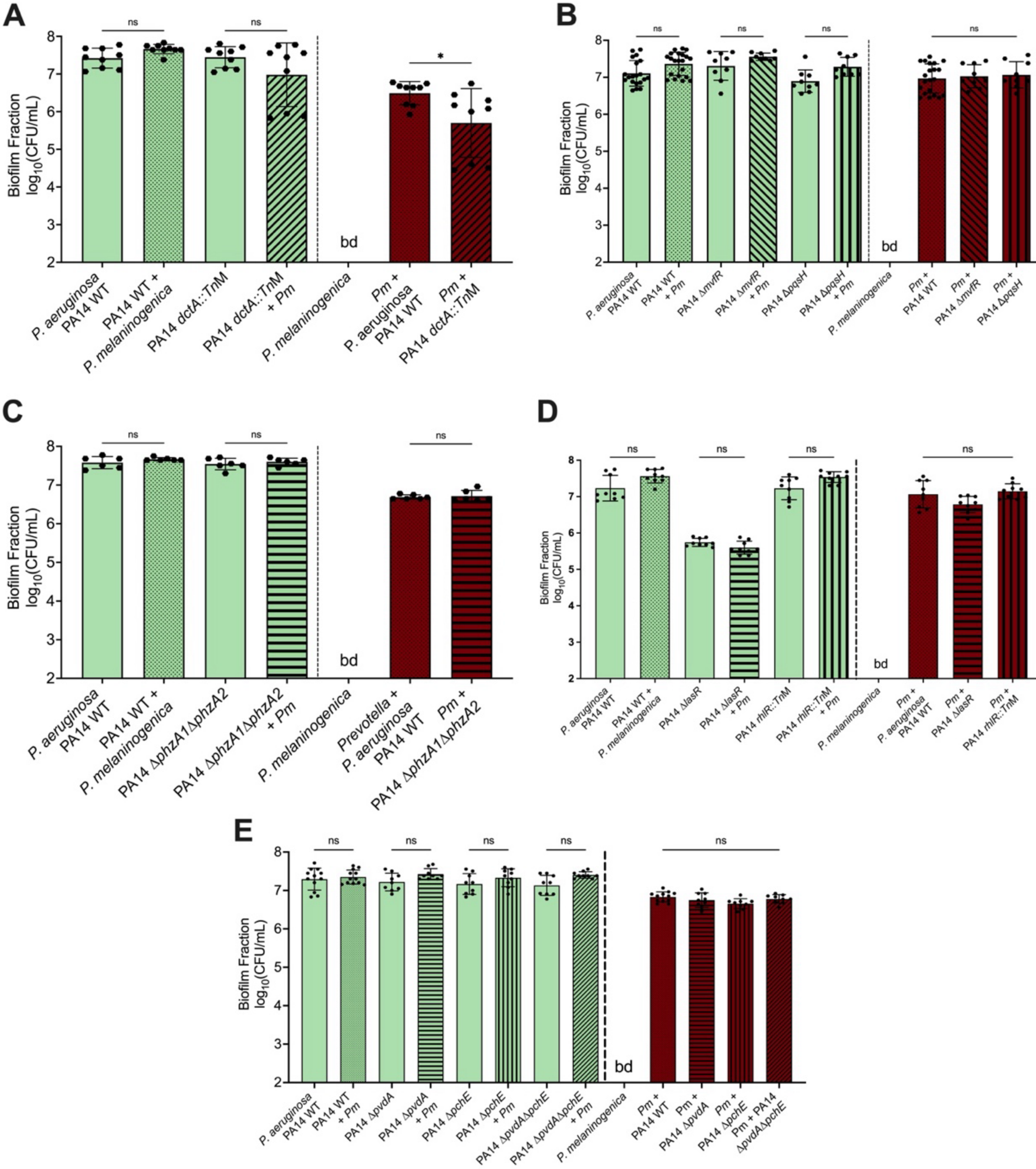
A
B
C
D
E
Biofilm Fraction
log10(CFU/mL)
ns
*
bd
P. aeruginosa PA14 WT
PA14 WT + P. melaninogenica
PA14 dctA::TnM
PA14 dctA::TnM + Pm
P. melaninogenica
Pm + P. aeruginosa PA14 WT
Pm + PA14 dctA::TnM
PA14 WT + Pm
PA14 ΔmvfR
PA14 ΔmvfR + Pm
PA14 ΔpqsH
PA14 ΔpqsH + Pm
Pm + PA14 WT
Pm + PA14 ΔmvfR
Pm + PA14 ΔpqsH
PA14 ΔphzA1ΔphzA2
PA14 ΔphzA1ΔphzA2 + Pm
Prevotella + P. aeruginosa PA14 WT
Pm + PA14 ΔphzA1ΔphzA2
PA14 ΔlasR
PA14 ΔlasR + Pm
PA14 rhlR::TnM
PA14 rhlR::TnM + Pm
Pm + PA14 ΔlasR
Pm + PA14 rhlR::TnM
PA14 ΔpvdA
PA14 ΔpvdA + Pm
PA14 ΔpchE
PA14 ΔpchE + Pm
PA14 ΔpvdAΔpchE
PA14 ΔpvdAΔpchE + Pm
Pm + PA14 ΔpvdA
Pm + PA14 ΔpchE
Pm + PA14 ΔpvdAΔpchE

Figure S6. The co-culture of *P. melaninogenica* and *P. aeruginosa* PA14 clean deletion mutants in candidate genes. A-E. All cultures were performed using mucin-containing ASM under anaerobic growth conditions at 37ºC for 24 hours. The biofilm fractions of the co-cultures are plotted. Statistical significance was calculated using ordinary one-way analysis of variance (ANOVA) with Tukey’s multiple comparisons test for all panels. **A.** The mono- and co-cultures of WT *P. aeruginosa* PA14 and the *P. aeruginosa* PA14 *dctA*::TnM mutant with *P. melaninogenica* (*Pm*). * $p < 0.05$. **B.** The mono- and co-cultures of WT *P. aeruginosa* PA14 and the ∆*mvfR* and ∆*psqH* mutants with *P. melaninogenica* (*Pm*). **C.** The mono- and co-cultures of WT *P. aeruginosa* PA14 and the ∆*phzA1*∆*phzA2* mutant with *P. melaninogenica* (*Pm*). **D.** The mono- and co-cultures of WT *P. aeruginosa* PA14 and the ∆*lasR* and *rhlR*::TnM mutants with *P. melaninogenica* (*Pm*). **E.** The mono- and co-cultures of WT *P. aeruginosa* PA14 and the ∆*pvdA,* ∆*pchE*, and ∆*pvdA*∆*pchE* mutants with *P. melaninogenica* (*Pm*).

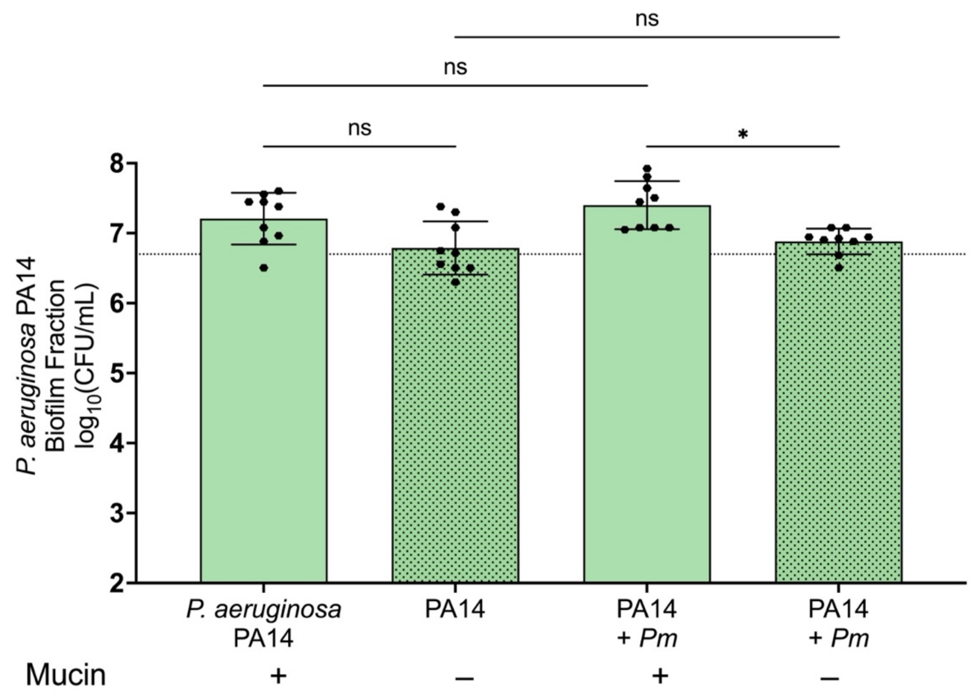

Figure S7. ***P. aeruginosa* grows well with or without mucin in ASM.** *P. aeruginosa* PA14 mono- and co-cultures with *P. melaninogenica* (*Pm*) in ASM with and without mucin under anaerobic growth conditions at 37ºC for 24 hours. Statistical significance was calculated using ordinary one-way analysis of variance (ANOVA) with Tukey's multiple comparisons test * $p < 0.05$.

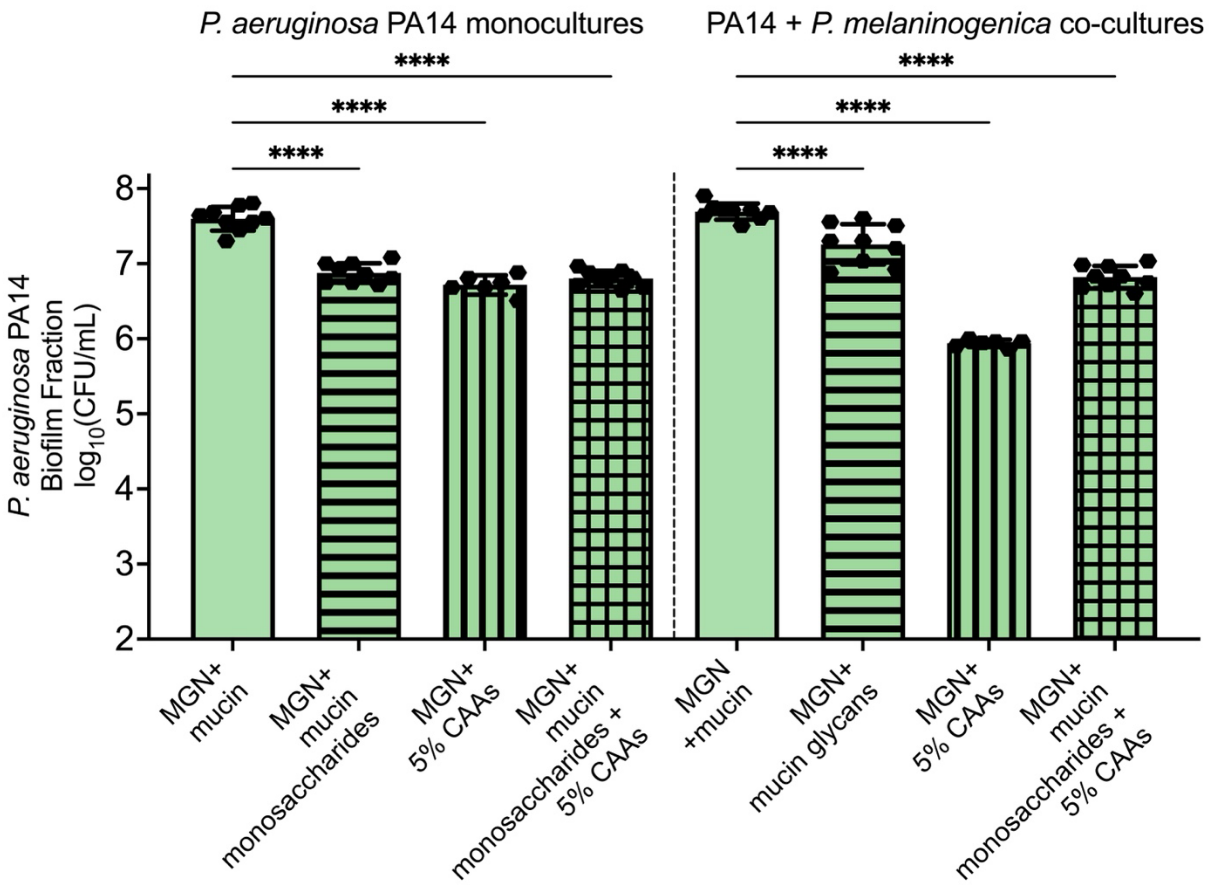

Figure S8. *P. aeruginosa* can grow in minimal medium with either mucin or mucin components. The *P. aeruginosa* PA14 mono- and co-cultures with *P. melaninogenica* using a minimal medium composed of M63 minimal salts base plus 0.2% glycerol and 100 μM nitrate, with the addition of either mucin or mucin components. The mucin components are mucin monosaccharides (galactose, fucose, N-acetylgalactosamine, and N-acetylglucosamine) or amino acids (CAAs – casamino acids). Statistical significance was calculated using ordinary one-way analysis of variance (ANOVA) with Tukey's multiple comparisons test. **** $p < 0.0001$.
