# Supplementary material for: Pseudomonas aeruginosa supports the survival of Prevotella melaninogenica in a cystic fibrosis lung polymicrobial community through metabolic cross-feeding
Source: mBio. 2025 Sep 12;16(10):e01594-25. doi: 10.1128/mbio.01594-25 (PMC12506151; doi:10.1128/mbio.01594-25)
Supplement: Table S1 — P. aeruginosa PA14 genes identified in the transposon mutagenesis screen. [file mbio.01594-25-s0002.pdf]

Supplementary Table S1. *P. aeruginosa* PA14 genes identified in the transposon mutagenesis screen.

The resulting list of *P. aeruginosa* PA14 transposon mutants that were incapable of supporting the growth of *P. melaninogenica* in co-culture in ASM following multiple confirmatory rounds of library screening.

PA14 Gene Locus	PAO1 Ortholog	GeneID	Gene Name	Gene Description	UniprotKB	SMART Protein Description
PA14_02590	PA0212	GID3016	*mdcE*	Malonate decarboxylase gamma subunit	A0A0H2ZKL8	Malonate decarboxylase subunit gamma
PA14_09810	PA4181	GID3354	-	Conserved hypothetical protein	A0A0H2ZG65	-
PA14_21150	PA3315	GID2932	-	Probable ABC transporter permease component	A0A0H2ZCQ8	-
PA14_21260	-	GID6316	-	Hypothetical protein	A0A0H2ZE37	Uncharacterized protein
PA14_27110	PA2861	GID4126	*ligT*	2'-5' RNA ligase	A0A0H2ZBJ6	RNA 2',3'-cyclic phosphodiesterase
PA14_31310	PA2568	GID4731	-	Hypothetical protein	A0A0H2ZBW8	Uncharacterized protein
PA14_36000	-	GID4903	*prpR*	Probable transcriptional regulator	A0A0H2ZAW3	PrpR, probable propionate catabolism operon regulator
PA14_37900	PA2057	GID124	*sppR*	Putative TonB-dependent receptor	A0A0H2ZAH5	Putative TonB-dependent receptor
PA14_38140	PA2040	GID995	-	Putative glutamine synthetase	A0A0H2ZA90	Putative glutamine synthetase
PA14_40930	PA1825	GID3669	-	Hypothetical protein	A0A0H2Z9R5	Uncharacterized protein
PA14_49930	PA1116	GID2795	-	Hypothetical protein	A0A0H2Z7Y8	Uncharacterized protein
PA14_53260	PA0851	GID2270	-	Hypothetical	A0A0H2Z710	Threonine dehydratase
PA14_54010	PA0791	GID2983	-	Probable transcriptional regulator	A0A0H2Z7C6	Transcriptional regulator, AraC family
PA14_59750	-	GID1058	*cupD4*	Fimbrial subunit	A0A0H2ZG53	Fimbrial subunit
PA14_63860	PA4830	GID4065	-	Hypothetical protein	A0A0H2ZH08	Thioesterase
PA14_64590	PA4887	GID1133	-	Putative MFS transporter	A0A0H2ZI81	MFS transporter
PA14_67350	PA5100	GID553	*hutU*	Urocanase	Q02ER6	Urocanate hydratase
