# Supplementary material for: Pseudomonas aeruginosa supports the survival of Prevotella melaninogenica in a cystic fibrosis lung polymicrobial community through metabolic cross-feeding
Source: mBio. 2025 Sep 12;16(10):e01594-25. doi: 10.1128/mbio.01594-25 (PMC12506151; doi:10.1128/mbio.01594-25)
Supplement: Table S2 — Strains and plasmids. [file mbio.01594-25-s0003.pdf]

Supplementary Table S2. List of strains and plasmids used in this study.

Strain	Genotype	Reference or Source
Prevotella melaninogenica ATCC 25845	WT	(1)
Staphylococcus aureus Newman	WT	(2)
Streptococcus sanguinis SK36	WT	(3)
Pseudomonas aeruginosa PA14	WT	(4)
Pseudomonas aeruginosa PA14	Δ*mdcA*	(5)
Pseudomonas aeruginosa PA14	Δ*mdcE*	
Pseudomonas aeruginosa PA14	Δ*mdcC*	
Pseudomonas aeruginosa PA14	Δ*mdcC::mdcC*	
Pseudomonas aeruginosa PA14	Δ*prpB*	(6)
Pseudomonas aeruginosa PA14	Δ*acsA*	
Pseudomonas aeruginosa PA14	Δ*prpB*Δ*acsA*	
Pseudomonas aeruginosa PA14	Δ*sucDC*	This study
Pseudomonas aeruginosa PA14	Δ*pauA*	
Pseudomonas aeruginosa PA14	Δ*prpB*Δ*mdcC*	
Pseudomonas aeruginosa PA14	Δ*sucDC*Δ*prpB*	
Pseudomonas aeruginosa PA14	Δ*sucDC*Δ*mdcC*	
Pseudomonas aeruginosa PA14	Δ*mdcC*Δ*pauA*	
Pseudomonas aeruginosa PA14	Δ*sucDC*Δ*prpB*Δ*mdcC*	
Pseudomonas aeruginosa PA14	Δ*prpB*Δ*mdcC*Δ*pauA*	
Pseudomonas aeruginosa PA14	Δ*prpB*Δ*mdcC*Δ*sdhBADC*	
Pseudomonas aeruginosa PA14	Δ*cbrA*	(7)
Pseudomonas aeruginosa PA14	Δ*cbrB*	
Pseudomonas aeruginosa PA14	Δ*crc*	
Pseudomonas aeruginosa PA14	*dctA::TnM*	(8)
Pseudomonas aeruginosa PA14	Δ*phzA1*Δ*phzA2*	(9)
Pseudomonas aeruginosa PA14	Δ*pvdA*	(10)
Pseudomonas aeruginosa PA14	Δ*pchE*	
Pseudomonas aeruginosa PA14	Δ*pvdA*ΔpchE	
Pseudomonas aeruginosa PA14	Δ*pqsR*	(11)
Pseudomonas aeruginosa PA14	Δ*pqsH*	

Plasmid	Reference or Source
pSMV8::*prpB*-KO	(6)
pEX18Gm::*sucDC*-KO	This study
pEX18Gm::*sdhBADC*-KO	
pMQ30::*pauA*-KO	

Literature Cited.

1. Shah HN, Collins DM. 1990. *Prevotella*, a new genus to include *Bacteroides melaninogenicus* and related species formerly classified in the genus *Bacteroides*. International Journal of Systematic and Evolutionary Microbiology 40:205–208.
2. Duthie ES. 1952. Variation in the antigenic composition of Staphylococcal coagulase. Microbiology 7:320–326.
3. Kilian M, Holmgren K. 1981. Ecology and nature of immunoglobulin A1 protease-producing *Streptococci* in the human oral cavity and pharynx. Infection and Immunity 31:868–873.
4. Rahme LG, Stevens EJ, Wolfort SF, Shao J, Tompkins RG, Ausubel FM. 1995. Common virulence factors for bacterial pathogenicity in plants and animals. Science 268:1899–1902.
5. Maderbocus R, Fields BL, Hamilton K, Luo S, Tran TH, Dietrich LEP, Tong L. 2017. Crystal structure of a Pseudomonas malonate decarboxylase holoenzyme hetero-tetramer. Nature Communications 8:160.
6. Flynn JM, Niccum D, Dunitz JM, Hunter RC. 2016. Evidence and role for bacterial mucin degradation in cystic fibrosis airway disease. PLOS Pathogens 12:e1005846.
7. Mould DL, Stevanovic M, Ashare A, Schultz D, Hogan DA. 2022. Metabolic basis for the evolution of a common pathogenic *Pseudomonas aeruginosa* variant. eLife 11:e76555.
8. Liberati NT, Urbach JM, Miyata S, Lee DG, Drenkard E, Wu G, Villanueva J, Wei T, Ausubel FM. 2006. An ordered, nonredundant library of *Pseudomonas aeruginosa* strain PA14 transposon insertion mutants. Proceedings of the National Academy of Sciences of the United States of America 103:2833–2838.
9. Dietrich LEP, Price-Whelan A, Petersen A, Whiteley M, Newman DK. 2006. The phenazine pyocyanin is a terminal signalling factor in the quorum sensing network of *Pseudomonas aeruginosa*. Molecular Microbiology 61:1308–1321.
10. Wang Y, Wilks JC, Danhorn T, Ramos I, Croal L, Newman DK. 2011. Phenazine-1-carboxylic acid promotes bacterial biofilm development via ferrous iron acquisition. J Bacteriol 193:3606–3617.
11. Cugini C, Morales DK, Hogan DA. 2010. *Candida albicans*-produced farnesol stimulates *Pseudomonas* quinolone signal production in LasR-defective *Pseudomonas aeruginosa* strains. Microbiology 156:3096–3107.
